# Mouse Models of Polyglutamine Diseases: Review and Data Table. Part I

**DOI:** 10.1007/s12035-012-8315-4

**Published:** 2012-09-07

**Authors:** Maciej Figiel, Wojciech J. Szlachcic, Pawel M. Switonski, Agnieszka Gabka, Wlodzimierz J. Krzyzosiak

**Affiliations:** Institute of Bioorganic Chemistry, Polish Academy of Sciences, Noskowskiego 12/14, 61-704 Poznan, Poland

**Keywords:** Polyglutamine, Mouse models, Huntington’s disease, Spinocerebellar ataxia, DRPLA, SBMA

## Abstract

Polyglutamine (polyQ) disorders share many similarities, such as a common mutation type in unrelated human causative genes, neurological character, and certain aspects of pathogenesis, including morphological and physiological neuronal alterations. The similarities in pathogenesis have been confirmed by findings that some experimental in vivo therapy approaches are effective in multiple models of polyQ disorders. Additionally, mouse models of polyQ diseases are often highly similar between diseases with respect to behavior and the features of the disease. The common features shared by polyQ mouse models may facilitate the investigation of polyQ disorders and may help researchers explore the mechanisms of these diseases in a broader context. To provide this context and to promote the understanding of polyQ disorders, we have collected and analyzed research data about the characterization and treatment of mouse models of polyQ diseases and organized them into two complementary Excel data tables. The data table that is presented in this review (Part I) covers the behavioral, molecular, cellular, and anatomic characteristics of polyQ mice and contains the most current knowledge about polyQ mouse models. The structure of this data table is designed in such a way that it can be filtered to allow for the immediate retrieval of the data corresponding to a single mouse model or to compare the shared and unique aspects of many polyQ models. The second data table, which is presented in another publication (Part II), covers therapeutic research in mouse models by summarizing all of the therapeutic strategies employed in the treatment of polyQ disorders, phenotypes that are used to examine the effects of the therapy, and therapeutic outcomes.

**Electronic supplementary material:**

The online version of this article (doi:10.1007/s12035-012-8315-4) contains supplementary material, which is available to authorized users.

## Introduction

The polyglutamine (polyQ) family of disorders comprises nine diseases, including Huntington’s disease (HD) [1]; spinal and bulbar muscular atrophy, X-linked 1 (SMAX1/SBMA) [2, 3]; six spinocerebellar ataxias (SCA) type 1 [4], type 2 [5], Machado–Joseph disease (MJD/SCA3) [6], type 6 [7], type 7 [8], and type 17 [9, 10]; and dentatorubral-pallidoluysian atrophy (DRPLA) [11, 12]. The polyQ diseases have multiple commonalities [13]. PolyQ diseases are all neurological disorders, and their pathology is mainly related to dysfunction or even a loss of neurons in the central nervous system (CNS). Recently, the non-neuronal pathology has been increasingly recognized and investigated. The polyQ diseases are caused by an unusual type of mutation, namely, the expansion of CAG repeats in the coding region of causative genes that are otherwise structurally unrelated. The presence of one mutant allele containing a CAG repeat number that exceeds a pathologic threshold is sufficient to cause the disease; therefore, all of these diseases are dominantly inherited. One exception is SMAX1/SBMA, which is linked to the androgen receptor (AR) gene on the X chromosome; thus, one affected allele in female patients may cause no (or only mild) symptoms compared to male patients.

In all of the polyQ diseases, the number of CAG repeats is dynamic and can expand or contract from generation to generation. The presence of a mutant allele leads to the production of a pathogenic protein containing an expanded polyQ tract that alters the properties and activity of the affected protein. The disease mechanism may include a gain and/or loss of function in the mutant protein, which becomes toxic to cells. The severity of the disease symptoms increases markedly with an increasing number of CAG repeats and the resulting number of glutamines in causative proteins; however, the naturally occurring low number of CAG repeats in the causative genes encodes polyQ stretches in their respective proteins without evoking any signs of a disease. Another feature that is common to the polyQ disorders is that many mouse models of polyQ diseases share common phenotypes, and this phenomenon resembles the situation in patients wherein many SCA disorders are clinically indistinguishable based on a neurological examination alone. These similar features led to the hypothesis that all polyQ disorders are in fact one disease that may have a common treatment. However, the extent to which this is true is limited because each disease possesses unique features as a result of the protein context of the polyQ mutation. Because the polyQ diseases (including their respective mouse models) are similar, understanding the mechanisms and pathogenesis of one disease or mouse model may contribute to our understanding of the mechanisms of other diseases.

**Fig. 1:**
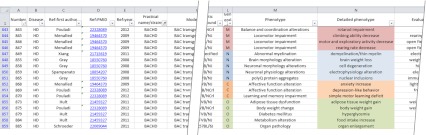
The data table is an electronic resource that collects the behavioral, molecular, cellular, and anatomic characteristics of polyQ mice. The figure demonstrates only a small fragment of the data table, and the selection of records for this figure is accidental. The full data table comprises over 2,000 records and 21 columns

The first transgenic animal that was generated as a model of a polyQ disorder and that showed the disease was the B05 SCA1 model mouse [14]. To date, more than 100 polyQ mouse models have been created, although some diseases are overrepresented (e.g., HD) [15] and others are underrepresented (e.g., SCA6) [16, 17] (Supp. Fig. 1). At this stage, finding, comparing, and translating the information between diseases are difficult without using special tools such as a database or sortable list. Therefore, we collected and integrated the body of data regarding disease onset and progression and therapeutic approaches in mouse models of the various polyQ diseases by creating an Excel data table (referred to as the data table). The data table is attached to this publication as supplementary material and covers the behavioral, molecular, cellular, and anatomic characteristics of polyQ mice in 21 columns and over 2,000 records (Fig. 1).

The structure of our data table comprises two informal groups of data. The first group of data consists of 11 columns that describe the general features of each mouse model, such as the disease that was modeled; references; the practical name of the mouse as used in the original publication; the model type; the genetic background of the mouse; and the data regarding the transgenic construct, including the promoter, transgenic sequence, and the number of CAG (Q) repeats within the structure of the transgene. The second group consists of nine columns that contain data characterizing the disease models by phenotypes divided into general phenotypes, phenotypes, detailed phenotypes, the methods that were used to detect the phenotypes, the affected brain regions or other tissues (if applicable), the age of the earliest and latest detection of the phenotype, and a quantification of the phenotype (if data are available) expressed as a percent decrease or increase relative to nontransgenic animals. The last column contains various comments that refine or add more details to other columns and provides additional references. Supplementary Table 1 summarizes the contents of the columns in the data table.

The data for the data table come from almost 290 carefully selected research articles that report the generation and/or characterization of transgenic animals. In this review, in addition to constructing the data table, we provide a “classic” text overview of polyQ mouse models. This review quotes a fairly complete set of scientific publications describing polyQ mouse model generation and characterization; however, many important works, particularly those in which no mouse models were directly investigated, were omitted due to space constraints. Therapeutic research using polyQ mouse models is included in our companion review (Part II), which summarizes the preclinical therapeutic strategies employed in polyQ disorders. We believe that this combination of text and table reviews can provide the most comprehensive overview of in vivo research on polyQ disorders.

Although the initial studies in which transgenic mice were created and characterized were very sparse and often inaccurate, the characterization of the disease phenotypes that are present in polyQ transgenic mice evolved rapidly as researchers learned what to look for in polyQ models. Unfortunately, the lack of guidelines, protocols or a systematic list of phenotypes that can aid in and standardize the characterization of polyQ mouse models remains a general issue within the field of polyQ and neurological disease research. A useful resource is the Mouse Genome Informatics (MGI) database (http://www.informatics.jax.org), which provides the Mammalian Phenotype Browser (MPB) [18]. The MPB contains very broad lists of phenotypes for all forms of disease models. The disadvantage of this solution is that it does not offer the subset of phenotypes that are specific to polyQ or neurological diseases. In addition, some phenotypes (such as the cognitive phenotypes observed in the R6/2 model) are not listed, and it contains only a partial list of transgenic animals and variants. For example, the MPB contains 7 models of SCA3, whereas our data table contains 14 mouse models and variants of SCA3. Additionally, our data table contains more precise information regarding phenotype timelines.

The current lack of both a coherent set of phenotypes and a list of polyQ mouse models delays the gathering of new information from mouse models, which makes it difficult to compare information among models and to compare information from mouse models with human patients. More precise characterization of polyQ disease models and the exchange of data from various disease models may accelerate the discovery of disease mechanisms and effective therapeutic strategies.

## Mouse Models of PolyQ Disorders

### HD Mouse Models

The *HTT* gene contains 67 exons spanning 169 kb and encodes a 3142-aa protein (huntingtin) from a 13-kb transcript. Because the CAG expansion is located in the first exon of the *HTT* gene, it is possible to generate simple transgenic models that express short fragments of the *HTT* gene containing the promoter, exon 1 and intron 1 [19, 20], short fragments of cDNA [21–24] or the full-length cDNA [25] driven by a non-native promoter. Other transgenic models have been generated by introducing the entire *HTT* gene with a yeast or bacterial artificial chromosome (YAC or BAC, respectively) [26–30]. Another strategy for overcoming the size of the *HTT* gene is using the mouse gene as a framework and introducing a CAG repeat tract [31] or CAG repeats in a chimeric human exon 1 [32–34] using a knock-in technique. In addition, both conditional and reversible HD models have been created to demonstrate the cell- and brain region-specific effects of mutant huntingtin or its fragments [35–37]. To date, the size of the *HTT* gene has hindered the creation of a “whole-gene-humanized” targeted knock-in mouse model of HD.

#### R6/2 Mouse Models

The first genetic model of HD was serendipitously created in 1996 by Bates and coworkers while studying the CAG instability in the *HTT* gene [19]. Three different transgenic lines— R6/1 (116 CAG), R6/2 (144 CAG) and homozygous R6/5 (128–156 CAG)—exhibit progressive neurological phenotypes. Because the R6/2 line shows instability of the repeat tract, variants containing 200–249, 250–299, 300–349, 350–399 and >400 CAG repeats have been created by breeding [38]. Interestingly, the disease is mitigated by an increase in the number of CAG repeats [38, 39].

The R6/2 line exhibits the most severe and rapid onset of motor and cognitive phenotypes among all of the available HD mice and is the most commonly studied model of this disease [40]. The R6/1 is a late-onset HD model [41] that often complements the use of R6/2 in therapeutic approaches (Part II).

Young R6/2 mice are indistinguishable from wild-type (WT) animals by home cage observation, but early tests reveal that the transgenic animals exhibit cognitive deficits as early as 3.5 weeks prior to the onset of overt motor abnormalities [42–46] (Table 5 and the data table). Cognitive deficits in R6/2 comprise spatial (hippocampal-dependent) and non-spatial (striatal-dependent) learning and memory, and such impairments are very similar to those observed in HD patients [46–50]. The motor abnormalities appear over the subsequent weeks, and their severity and frequency increase until they finally develop into the evident neurological signs of repetitive movements, wide-based gait, epileptic seizures and sudden involuntary movements of the whole body that may be the mouse equivalent of the chorea observed in HD patients [19, 42, 44, 45]. In addition, progressive body-weight loss is observed until death, which occurs prematurely between 13 and 15 weeks of age [44], but the lifespan of the animal increases with an increasing number of CAG repeats [38, 39].

The neurodegeneration in R6/2 mice has been intensively studied, revealing pathological changes in neuronal morphology, such as the presence of nonapoptotic dark neurons in specific areas of the brain [51, 52]. Striatal medium-sized spiny neurons (MSNs) exhibit electrophysiological changes in addition to altered morphology [53–55]. The models of polyQ diseases and patients contain neuronal intranuclear inclusions (NIIs) that can also be identified in R6/2 as early as postnatal day 1 in various brain regions [44, 56–58]. The R6/2 brain progressively loses weight [44, 59, 60], but not as a result of neuronal loss in the striatum, because neuronal death was not observed [52, 61]. However, Stack and colleagues [44] observed a 25 % reduction in the number of striatal neurons and also detected reactive gliosis in R6/2 animals. This finding was later confirmed by another group [62]. Despite these results, the findings from R6/2 mice largely support the hypothesis that neuronal dysfunction (rather than neuronal loss) is responsible for the onset of neurological phenotypes. If the mice were to live beyond 13 weeks of age, they may eventually show a larger reduction in the number of MSNs.

**Fig. 2:**
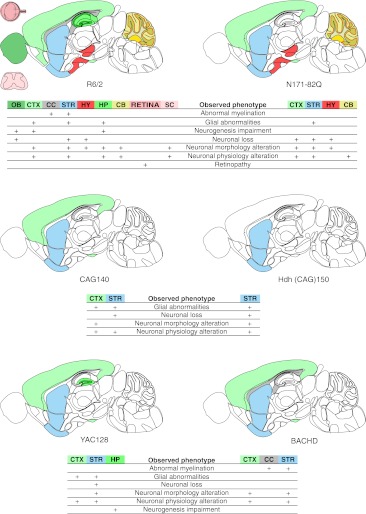
Diagrams of neuropathology in the brain, spinal cord, and retina of selected mouse models of Huntington’s disease. Seven neuropathology phenotypes, namely, neuronal loss, neuronal morphology alterations, neuronal physiology alterations, glia abnormalities, retinopathy, neurogenesis impairment, and abnormal myelination, were selected, along with the localization available for these phenotypes in CNS region column of the data table. The brain regions involved in the selected neuropathology phenotypes for R6/2, N171-82Q, CAG140, Hdh(CAG)150, YAC128, and BACHD models were marked with colors on a schematic sagittal section of the mouse brain. Brain regions were color-coded using colors from the Allen Brain Atlas [374]. The main brain regions involved in the HD models are the striatum (*STR*) and cerebral cortex (*CTX*). The R6/2 and YAC128 models also show involvement of the hippocampus (*HP*) as a region of “neurogenesis impairment” [375, 376]; the deficit is also observed in the piriform cortex (*CTX*, *bottom part*) [377] and olfactory bulb (*OB*) [378] of the R6/2 mice. Moreover, R6/2 neuropathology is observed in the hypothalamus (*HY*), cerebellum (*CB*), spinal cord (*SC*), and retina of the eye (*RETINA*). The cerebral cortex (*CTX*) is not involved in the selected neuropathology phenotypes in the Hdh(CAG)150 knock-in. As in R6/2 animals, abnormal myelination has been detected in the corpus callosum (*CC*) and striatum (*STR*) of BACHD animals

Mutant huntingtin is also expressed in the retinas of R6/2 and R6/1 mice, which leads to pronounced vision deficiencies and retinal degeneration [63]. This huntingtin effect cannot be observed unless the background strain does not contain the *rd* mutation responsible for retinal degeneration (see “Mouse genetic background”). A map of the neuropathology of R6/2 mice can be found in Fig. 2.

R6/2 mice also exhibit severe non-neurological abnormalities, which are summarized in detail in Table 6 and the data table. Like HD patients, some R6/2 mice develop diabetes [64–66] and insulin resistance as a consequence of hypothalamic–pituitary–adrenal axis dysregulation [67]. Moreover, R6/2 mice exhibit cardiac dysfunction, which leads to severe cardiac failure; this finding is consistent with the early onset cardiovascular disease that occurs in HD patients [68]. Another non-neurological abnormality in R6/2 mice, which makes them difficult to maintain, is the prominent atrophy of their reproductive organs [19]. In addition, the circadian rhythms of R6/2 mice are altered [69, 70].

Taken together, the R6/2 models have demonstrated the multitude of phenotypes that can also be detected in HD patients and have established the role of mutant huntingtin exon 1 in the pathogenesis of HD.

#### N171-82Q Mouse Models

The N171-82Q transgenic model, which expresses the N-terminal fragment of the human huntingtin protein, was created in Borchelt’s laboratory [21]. The transgene molecule comprises the first 171 amino acids, but it contains a stretch of 82 glutamines instead of the 23 glutamines that are present in the reference sequence [21, 71].

N171-82Q mice exhibit a neurological phenotype that is rapidly progressive but slower than that of R6/2 animals. The disease progresses to the evident loss of coordination, hypokinesis, motor and learning deficits, and it ends in premature death [21, 72–74].

As in the R6/2 mice, the NIIs of mutant huntingtin are present in neurons in many brain regions (see the data table) [21, 52, 75, 76]. N171-82Q mice exhibit progressive neurodegeneration that manifests in brain atrophy [77–80] and reactive gliosis [52]. Importantly, the degenerating neurons in N171-82Q animals die from apoptotic processes [52], whereas apoptosis is not detected in degenerating neurons of R6/2 mice. Another marked difference from the R6/2 model is the evident loss of medium spiny neurons in the striata [80, 81]. However, the spectrum of disease phenotypes in N171-82Q mice is generally in line with those in the R6/2 mice. Because the transgene and protein are longer in N171-82Q mice, the model contributed to the discussion about the significance of transgene and protein length, as well as the putative cleavage of huntingtin in the pathogenesis of HD [71]. A map of the neuropathology of N171-82Q mice can be found in Fig. 2.

#### Transgenic YAC HD Models

A range of HD mouse models was produced in Hayden’s laboratory by introducing the human *HTT* gene using YAC. The models are named after the number of consecutive CAG repeats in the *HTT* gene and include the YAC18, YAC46, YAC72, and YAC128 lines [28]. YAC72 mice exhibit a mild disease course and also show NMDA receptor (NMDAR) abnormalities and impairment of mitochondrial metabolism [29, 82–85]. The YAC128 model was generated to further investigate HD and to produce a more severe disease phenotype than that of the YAC72 model [30]. YAC128 mice exhibit a progressive deterioration in rotarod performance, a biphasic activity profile that manifests as a transition from a hyperkinetic to a hypokinetic phenotype (detailed onset and progression of disease in YAC128 mice listed in Table 5 and the data table) [86, 87]. Cognitive abnormalities manifest as cognitive rigidity, as well as spatial and procedural learning deficits; therefore, both the hippocampal and striatal memory systems are affected. These changes precede the onset of motor deficits and resemble the cognitive impairments seen in patients [88–91].

In YAC128 animals, neurodegeneration is detected late, but the loss of striatal and cortical neurons is evident [30, 86, 92, 93]. The neuronal NIIs, as well as the extranuclear inclusions, in YAC128 mice are visible by light microscopy well after the development of the histological and behavioral abnormalities [20, 94]. For a map of neuropathology in the YAC128 model, see Fig. 2.

A long-debated feature of nearly every HD model is the resistance to excitotoxicity following the injection of quinolinic acid (QUIN). Interestingly, in the YAC models, the animals first develop an increased susceptibility to excitotoxicity and later develop a resistance to it. This is in contrast to the fast-occurring resistance seen in the R6/2 and N171-82Q models [95–100]. The transition from susceptibility to resistance most likely results from progressing neuronal dysfunction in the cortex and striatum that finally leads to a severe loss of connectivity between these brain structures [101–106]. It is possible that increased NMDAR-mediated currents in the MSNs compensate for a mild loss of corticostriatal connectivity during the early stage of HD in YAC128 mice. The increased currents in the MSNs probably result from elevated NMDAR protein levels [84, 107], which may be the reason behind the increased susceptibility to excitotoxicity after challenging the YAC128 brains with QUIN. The later resistance may develop after severe loss of corticostriatal connectivity, loss of spines, and reduction in the level of NMDAR expression. This problem is also discussed in our Part II review.

#### Knock-in HD Models

The R6 and N171-82Q models exhibit a severe, highly progressive HD phenotype but do not always reflect the real expression pattern of human *HTT* or the underlying molecular basis of the disease. To address this issue, knock-in mice with a wide range of CAG repeats (50–200; see the data table) were created. For instance, the 50, 92, and 111 CAG stretches were knocked-in by replacing exon 1 of the mouse *Htt* gene with a hybrid mouse/human sequence [108, 109]. HdhQ92 and HdhQ111 mice have both intergenerational and somatic repeat instability [109], showing a very mild phenotype that appears almost exclusively when homozygous [110, 111]. Although the deficits in the acoustic startle response and cognitive deficits are detected around 4 months, motor impairments were not detected before 24 months [32, 45, 112]. The cognitive deficits include changes in procedural learning and alternation learning [110, 113–116]. The NIIs are detectable in various brain regions and are composed of the N-terminal fragments of the HTT protein [117, 118]. Similar knock-in CAG71 and CAG94 animals with mild phenotypes were created by Chesselet’s group [119, 120] and have been recapitulated by the creation of a CAG140 model [33] in which the course of the disease is slightly accelerated. The disease progression is somewhere between those of the BACHD and YAC128 models, and the model also exhibits the biphasic behavior common for many HD models (Table 5 and the data table). The mice have abnormal balance and coordination [33, 121–123] as well as increased anxiety and motor learning deficits. At the level of the brain tissue, a reduction in striatal volume, the loss of striatal neurons and the presence of NIIs in striatum parallel a progressive reduction of DARPP-32 expression in the MSNs [33, 122]. A map of the neuropathology of CAG140 mice can be found in Fig. 2.

A two-step gene targeting strategy was used by Detloff’s group to create HD knock-in Hdh(CAG)150 mice with CAG expansions that were inserted into the pure mouse *Htt* gene sequence [31]. Longitudinal studies of homozygous animals revealed that this model has a mild phenotype characterized by late-onset decreases in motor activity [124] along with cognitive deterioration [125, 126] and neuropathology [124, 127]. Recently, a knock-in HdhQ200 mouse with 200 CAG repeats was created that presents a slightly accelerated phenotype compared to that of the Hdh(CAG)150 mice [128].

#### Transgenic BACHD Model

The BACHD model was created using the entire human HD gene located in a 250-kb genomic fragment modified by inserting a CAA/CAG mixed sequence encoding the tract of 97 glutamines [27]. Unlike the pure CAG sequence, the CAA/CAG mixed sequence is genetically more stable and therefore suitable for testing whether the somatic instability of CAG repeats is essential for pathogenic processes in HD. Indeed, the BACHD mice show the same stable length of the mixed CAA/CAG tract in both maternal and paternal transmission, as well as in aged brain regions, including the striatum, cortex, and cerebellum. However, the stability of the mixed CAA/CAG tract does not prevent pathogenesis in the BACHD mice, and neuronal dysfunction clearly occurs long before neuronal degeneration is observed. For instance, the motor deficits are evident as early as 4 weeks, and increased anxiety manifests at 12 weeks of age [27, 45]. Although the neuropathological changes in electrophysiology can also be identified at 12 weeks [129, 130], the evidence of neurodegeneration is observed at 12 months in the form of atrophy of the cortex and striatum. As in the R6/2 mice, the BACHD mice have neuronal dark cell degeneration in the striatum [27]. Interestingly, the BACHD animals essentially lack NIIs; instead, they show the presence of extranuclear aggregates (neuropil aggregates) in the cortex and the striatum [27]. A map of the neuropathology of BACHD mice can be found in Fig. 2.

Information about other HD models that are not directly reviewed in the text is provided in Table 1.

**Table 1:** Other transgenic mouse models of HD

Model name	Promoter	CAG number	Properties	References
D9-N171-98Q	mDarpp-32	98	Cell-autonomous MSNs dysfunction, mild phenotype	[23]
HD48, HD89	CMV	48, 89	Full HTT cDNA; relatively slow, biphasic phenotype; decreased lifespan; neuronal loss, clasping and circling behavior	[25, 300]
HD 100	rat Eno2	100	Neuron-specific model; corticostriatal pathway impairment	[273, 313]
HD150QG, HD 190QG	HTT (1 kb of 5′UTR)	150, 190	EGFP-HTT model; R6-like phenotype; decreased life span; possible attenuating effect of eGFP on disease severity	[311]
NLS144, NES144	HTT (1 kb of 5′UTR)	143	Modified R6/2 constructs with either NLS or NES signals produce accelerated and ameliorated phenotypes, respectively	[276]
Htt-160Q	GFAP (Gfa2)	160	Glia-specific expression of N-terminal HTT; decreased lifespan; non-cell-autonomous mechanism of HD pathogenesis	[231]
NLS-N171-82Q	PrP	82	NLS signal ameliorates the phenotype of N171 mice	[22]
N118-82Q	PrP	82	Shorter N-terminal HTT fragment; N171-like phenotype with earlier premature death	[24]
N586-82Q/K14-eGFP	PrP	82	N-terminal HTT (equivalent to caspase-6 clevage product); phenotype milder than N171 with unexpected cerebellar pathology, dyskinesia and ataxia; frequent cytoplasmic aggregates	[236]
N586-82Q	PrP	82	N-terminal HTT (equivalent to caspase-6 clevage product)	[237]
shortstop	HTT	120	N-terminal YAC model; no disease phenotype; NIIs present	[20]
BACHD-SD, BACHD-SA	HTT	97^a^	Mutation of HTT at serines 13 and 16 to either phosphomimetic (SD) or phosphoresistant (SA) version; mice with normal and BACHD-like phenotype, respectively	[239]
HD94	CamKIIα	94	Forebrain-specific conditional (dox-regulated) model; reversal of NIIs and HD-like phenotype upon HTT expression turn off	[35]
Prp-tTA-6/iFL148Q-69	PrP	148	Conditional model; full-length HTT; phenotype similar to other N-terminal models, but slower disease progression rate; decreased lifespan	[37]
RosaHD/Dlx5/6-Cre	Rosa26/Dlx5/6	103	Restriction of HTT expression to MSNs; changes in NMDA receptor-mediated currents; no other neurological phenotypes	[233]
RosaHD/Emx1-Cre	Rosa26/Emx1	103	Restriction of HTT expression to cortical pyramidal neurons; no neurological phenotype; polyQ nuclear accumulation	[36]
RosaHD/Nestin-Cre	Rosa26/Nestin	103	Restriction of HTT expression to neurons; evident neuropathology; locomotor activity decrease	[36]

^a^Mixed CAA/CAG repeats

### SCA1 Mouse Models

#### SCA1 Pcp2/L7 Promoter-Based Models

The SCA1 mouse model (called B05) was created using the full-length *ATXN1* cDNA with 82 CAG repeats. The Purkinje cell (PC) protein 2 (*Pcp2*/L7) promoter limits the transgene expression to cerebellar PCs. The ataxin-1 mRNA content is massive due to the occurrence of approximately 30 copies of the transgene at a single integration site [14]. Voluminous but cell-population-limited transgene expression results in the close reproduction of the ataxic phenotype in mice but the absence of a decreased lifespan [131]. Cerebellar neuropathology develops prior to the onset of ataxia and involves strong and unusual morphological [131] and electrophysiological alterations in PCs [132, 133], as well as the presence of NIIs in 90 % of PCs [134]. Morphology alterations are accompanied by strong gliosis and the loss of PCs [131]. The B05 mice also exhibit changes in cerebellar gene expression [135] and signaling [136] as well as biochemical changes, similar to those of SCA1 patients [137].

A limitation of the B05 model is the use of the *Pcp2* promoter, which induces an ataxin-1 protein expression pattern that is inconsistent with the expression pattern of the native protein. High overexpression of the mutant protein in PCs results in abnormalities such as vacuolization and heterotopy of PCs; these features are not present in SCA1 patients.

The variants of the B05 model enhanced our knowledge of the mechanisms of SCA1 by elucidating the role of ataxin-1 phosphorylation at S776 [138, 139] and the role of the ataxin-1 NLS signal in the disease pathogenesis [140, 141]. The use of the conditional B05 variants also confirmed that the SCA1-like phenotype can be reversed upon doxycycline treatment [142–144].

#### SCA1 Knock-in Model

Knock-in models of SCA1 were created by the targeted insertion of chimeric mouse/human exon 8 with 78 and 154 CAG repeats into one allele of the *Atxn1* locus [145–147]. Mice containing 78Q in ataxin-1 develop neither ataxic behavior nor a neuropathological phenotype [145]. In contrast, mice containing 154Q (*Sca1* ^154Q/2Q^) develop ataxia that resembles SCA1 in human patients. Other phenotypes of *Sca1* ^154Q/2Q^ mice include growth retardation, muscle atrophy, kyphosis, and clasping. In addition, the cognitive deficits in *Sca1* ^154Q/2Q^ occur before the onset of ataxia and are likely associated with hippocampal dysfunction [146, 148]. The progressive respiratory dysfunction, which is also observed in human patients, is probably the direct cause of premature death in these mice [149].

**Fig. 3:**
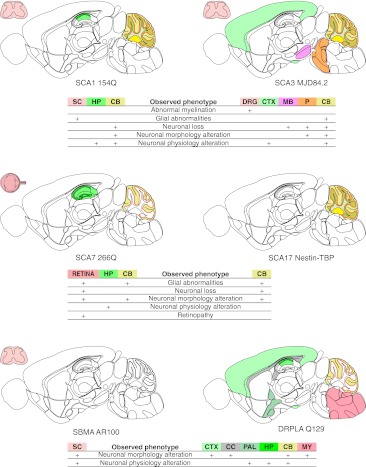
Diagrams of neuropathology of the brain, spinal cord, and retina in polyQ models that use native promoters. As in Fig. 2, the selected phenotypes and brain regions were marked with colors on a schematic sagittal section of the mouse brain. The polyQ models selected for this figure were the SCA1 154Q, SCA3 MJD84.2, SBMA AR100, DRPLA Q129, and SCA7 266Q models that were created using the mouse or human versions of native promoters. The exception to this scheme is the SCA17 nestin-TBP model, in which the native TBP promoter drives expression only in neurons. The SCA3 MJD84.2 and DRPLA Q129 models with native promoters demonstrate the involvement of various and partially overlapping brain regions. The neuropathology is localized in the cerebral cortex (*CTX*), pons (*P*), midbrain (*MB*), and cerebellum (*CB*) for MJD84.2 animals and in the cerebral cortex (*CTX*), pallidum (*PAL*), hippocampus (*HP*), medulla oblongata (*MY*), corpus callosum (*CC*), and cerebellum (*CB*) for the DRPLA Q129 model. These neuropathology patterns also resemble the patterns that are present in human patients. Abnormal myelination in MJD84.2 animals is observed in the dorsal root ganglia (*DRG*) and sciatic nerve, both of which belong to the peripheral nervous system (*PNS*)

The NIIs are formed more frequently throughout the brains of *Sca1* ^154Q/2Q^ mice than in SCA1 patients, probably because of the much longer polyQ tract in the mouse transgene. Interestingly, neurons that are less affected or unaffected exhibit more NIIs and gain NIIs at a faster rate [146]. The mice show pronounced brain atrophy, as well as the loss and degeneration of PCs [150]. However, in contrast to the B05 model, no vacuoles or heterotopic PCs can be detected [146]. Figure 3 contains a map of the neuropathology of *Sca1* ^154Q/2Q^ model.

### SCA2 Mouse Models

Cell-specific transgenic models of SCA2 were created using the *Pcp2* (L7) promoter-driven full-length *ATXN2* cDNA that contained 58 (58Q-5B) or 22 (22Q) CAG repeats. The 58Q-5B mice show mild and slowly progressing neurobehavioral dysfunctions and neuropathology including deficits in walking and coordination as well as the presence of clasping behavior [151]. The neuropathology involves the atrophy and loss (50 %) of PCs [152]. The mice show ubiquitin-negative microaggregates in the cytoplasm, but NIIs are not observed [151].

Another SCA2 model (75Q-SCA2) was created using the full-length human ataxin-2 cDNA driven by the human *ATXN2* promoter and containing 75 CAG repeats. The expression of this transgene is ubiquitous and is detected in the brain, liver, and skeletal muscle. The phenotype appears to be slightly accelerated compared to that of the 58Q-5B mice [153].

### SCA3 Mouse Models

#### SCA3 Pcp2/L7 Promoter-Based Models

The first attempt to create SCA3 transgenic mice revealed that using the *Pcp2* (L7) promoter and full-length *ATXN3* 79 CAG cDNA does not result in a disease phenotype in the PCs. Surprisingly, the presence of the C-terminal part of mutant ataxin-3 containing 79Q in the PCs was able to evoke a severe ataxic phenotype and cerebellar degeneration. This difference in phenotypes between the full-length and truncated ataxin-3 lines is probably due to a lack of ataxin-3 polyQ cleavage in the PCs [154].

A similar model was created by Torashima et al. [155] in which a C-terminal ataxin-3 fragment (exons 10-11 of *ATXN3* mRNA) containing 69 CAGs driven by the *Pcp2* (L7) promoter is expressed. The mice exhibit a neurobehavioral phenotype consisting of ataxia and cerebellar atrophy with heterotopic positioning of the PCs in the molecular layer and a shrunken dendritic tree.

#### SCA3 PrP Promoter-Based Models

The prion protein (PrP) promoter is employed in SCA3 models that express the full-length *ATXN3* cDNA but contain various numbers of CAG repeats. The first PrP/SCA3 hemizygous mouse with ataxin-3 containing 71 glutamines (the Q71-B and Q71-C mouse lines) exhibited no phenotype. A phenotype that is reminiscent of SCA3 appears in homozygous transgenic animals, but homozygosity renders the animals infertile [156, 157]. The homozygous mice show posture abnormalities, muscle wasting, seizures, and progressive, ataxia-like motor dysfunction [156]. The Q71-C homozygotes mice show no neuronal loss in the dentate nuclei but have a loss of tyrosine hydroxylase positive neurons only in the substantia nigra [156]. Notably, the MJD patients show severe neuronal loss in various brain regions, and this feature is better reproduced in the MJD-84.2 YAC model (see below and in the data table). The apparent reason for the discrepancy between the phenotypes of the hemizygous and homozygous mice is the difference in the concentrations of the toxic mutant ataxin-3 cleavage fragment in the brain [156]. In an attempt to prevent the cleavage of toxic polyQ from ataxin-3, the authors created the Q71 transgene, which lacks residues 190–220. Unfortunately, these deltaQ71 mice have both the cleavage fragment and exhibit an accelerated disease progression with an earlier premature death compared to the Q71 mice [158].

Another group created animals similar to Q71 with 15, 70 (70.61 mouse line), and 148 polyQ tracts in ataxin-3, but with a shorter version (1.1 kb) of the murine PrP promoter. The 70.61 line develops a mild to severe phenotype with ataxia and premature death [159]. Interestingly, later studies of the 70.61 line report a much milder phenotype and no reduction in the lifespan of the animals [160]. In the 148Q lines, the disease progression leads to decreased fertility and the death of all animals by 3 months of age. In both the 70.61 and 148Q lines, ubiquitin-positive inclusions are seen in many brain regions.

The subcellular localization of mutant ataxin-3 is an important aspect in the disease pathogenesis. Mice that express the nuclear export signal (NES)-tagged 148Q protein have normal lifespans with either nonexistent or very mild phenotypes. In contrast, the nuclear localization signal (NLS)-tagged 148Q mice exhibit a more severe phenotype than the 148Q mice [159]. Interestingly, the NES construct was unable to prevent the neurological phenotype in a double transgenic crossbreed of 70.61/NES.148 mice, which exhibit an aggravated ataxic phenotype [161].

Mutant ataxin-3 with 79 CAG repeats driven by the PrP promoter also results in a late phenotype in mice. The transgene is expressed in the brain areas that are involved in SCA3 pathology but not in PCs [162, 163].

#### The MJD-84.2 YAC Model

The mouse model that most closely mimics SCA3 was created using a 250-kb YAC construct that contains 50 kb of the human *ATXN3* gene flanked by 30- and 170-kb genomic sequences [164]. This indicates that MJD-84.2 YAC may in fact be transgenic for other human genes, and the possible candidates are TRIP11 and NDUFB1. The animals that were generated include lines with 15, 67, 72, and 84Q that vary in the age of onset and the severity of disease but generally present mild phenotypes. The neurobehavioral dysfunctions that are observed in the lines include an abnormal gait (mild to severe), mild tremor, hypoactivity, and limb clasping [164]. The pattern of neuropathology primarily includes NIIs formation, neuronal loss and gliosis of the dentate nucleus, pontine nuclei, and substantia nigra, along with visible myelination defects in peripheral nervous system [164, 165]. The cerebellar cortex, which is less affected in SCA3 patients than in SCA1 patients, is atrophic with Purkinje cell loss, dysfunction, and degeneration [164, 166]. Figure 3 contains a map of the neuropathology of MJD84.2 mice.

#### Other SCA3 Models

In contrast to PrP/ataxin-3 with 148Q [164], the mice that express ataxin-3 with a 148Q tract driven by the huntingtin promoter show only mild behavioral and motor changes [167]. A mild phenotype without an altered lifespan has been observed in mice expressing 94Q or 83Q in ataxin-3 driven by the cytomegalovirus (CMV) promoter [168]. Our group has attempted to generate a SCA3 knock-in model, but a functionally inactive allele was generated in the mice instead, resulting in a functional ataxin-3 knock-out [169].

### SCA6 Mouse Models

Creating a SCA6 knock-in mouse model may be challenging because the mouse homolog of the human calcium channel gene *CACNA1* lacks a CAG tract. The first attempt to generate a knock-in model of SCA6 (Q13/Q28) produced mice that were viable and healthy [16]. Thus, the range of expansions that causes SCA6 in human patients is probably insufficient for mice. Next, Watase et al. [17] created a model using targeted insertions of 84Q together with their flanking sequence into exon 47 of the mouse *Cacna1* gene. The heterozygous Sca6^84Q/+^ and homozygous Sca6^84Q/84Q^ are only mildly affected, with no detected neuropathology except for the presence of cytoplasmic inclusions in the PCs of 22-month-old Sca6^84Q/84Q^ mice and decreased Ca_v_2.1 protein expression that is accompanied by a reduced calcium current. The presence of the reduction in the Ca^2+^ current is not dependent on the number of CAG repeats in *Cacna1* because a reduced Ca^2+^ current is also evident in homozygous animals containing 14Q and 34Q [17].

### SCA7 Mouse Models

SCA7 is a very rare disease, but its modeling in mice generates systemic and complex phenotypes that reveal information significant for the entire polyQ research field. Moreover, the SCA7 mice provide an important model for polyQ nonautonomous pathogenesis of neurons, revealing the role of astrocytes and glutamate toxicity.

#### Cell-specific SCA7 Models with Pcp2/L7, Rhodopsin, and GFAP Promoters

The first SCA7 mice were cell-specific models that expressed an expanded ataxin-7 protein that contained a 90Q expansion either in the PCs using the *Pcp2* promoter (strain P7E) or exclusively in the retina using the rhodopsin promoter (strain R7E) [170]. P7E.B mice contain 10–50 copies of the transgene and show only some defects in motor performance and learning. In this model, human ataxin-7 translocates to the nucleus of the PCs [171] and eventually forms large perinucleolar aggregates. The neuropathology involves the degeneration of PCs and deep cerebellar nuclei [170]. R7E transgenic mice develop a phenotype earlier than P7E mice, with a pathology that includes NII formation, retinal photoreceptor dysfunction, and neurodegeneration [170, 172–174]. One possible reason for the retinopathy in these mice is the toxicity of the expanded polyQ tracts that deregulate rod differentiation genes [175].

The GFAP (Gfa2) promoter [176] restricts the expression of a transgene to astrocytes within the brain, including the cerebellar subpopulation of astrocytes called Bergmann glia. A Gfa2-SCA7-90Q mouse model expressing ataxin-7 with a 90Q exhibits an ataxic phenotype and PC degeneration [177]. The mice develop an abnormal gait and poor coordination but do not die prematurely. Neuropathological analysis revealed dark cell degeneration of PCs and the formation of NII and abnormal radial processes in the Bergmann glia. Dark cell degeneration is indicative of glutamatergic excitotoxicity, which corresponds to the 20–25 % decrease in glutamate uptake that is observed in cerebellar slices from Gfa2-SCA7-90Q mice. This decrease in glutamate uptake likely results from the dysfunction of the Bergmann glia, which express the GLT-1 and GLAST glutamate transporters. The authors postulated that the non-cell-autonomous, excitotoxicity-mediated pathology of PCs leads to the development of ataxia in SCA7 [177]. Thus, a potential therapeutic strategy for SCA7 (and also for HD) is to increase the expression of glutamate transporters in the brain. We have identified PACAP, TGF-α, and EGF as potent positive regulators of both GLAST and GLT-1 glutamate transporters [178, 179].

#### SCA7 PrP Promoter-Based Models

The PrP-SCA7-c92Q is the PrP polyQ model, expressing human *ATXN7* cDNA with 92 CAG repeats [180]. The animals show smaller body size, clasping, and premature death, and these features are characteristic for other PrP polyQ models. These mice are well-characterized and present an interesting systemic phenotype that includes ataxia, retinopathy, motility dysfunctions, non-cell-autonomous neurodegeneration of PCs and Bergmann glia pathology [181–183].

#### SCA7 Knock-in Model

Knock-in SCA7 266Q/5Q mice were created by inserting the human exon 3 of *ATXN7* containing 266 CAG repeats [184], which is in the repeat number range that is present in patients with juvenile-onset SCA7. Similar to pediatric patients, the mice develop a rapidly progressive complex phenotype that includes ataxia, myoclonic seizures, strong retinopathy leading to blindness and additionally posture abnormalities and, later, infertility. In the end stage of the disease, the mice are hypoactive and die at 19–20 weeks of age (7–8 weeks as homozygous) [184]. Interestingly, despite the ubiquitous expression of ataxin-7 266Q, the only abnormal neuronal morphology in the cerebellum is a decrease in the sizes of the PCs, whereas the dendritic trees and number of cells appear to be normal [184, 185]. More severe abnormalities are observed in the Bergmann glia, which also express the mutant ataxin-7 [177] (for a map of neuropathology, see Fig. 3). Aggregates are present in the glial cells and later in the PCs and hippocampal CA1 pyramidal neurons. In the retina, the inclusions appear first in the cones and interneurons of the outer and inner nuclear layers, respectively, and later in the rods and ganglion cells [173, 184].

The SCA7 models that utilize the platelet-derived growth factor (PDGF) promoter [171, 186] and the BAC models [187] are listed in the data table.

### SCA17 Mouse Models

The first SCA17 mouse model expresses the full-length human TATA-box binding protein (hTBP) with either 71 or 105 CAG/CAA repeats in the mRNA under the control of the PrP promoter [188]. The phenotype is observed early and featured by low weight, ataxia, and a reduced lifespan. The mice are hypoactive and develop seizures, tremors and kyphosis. The hTBP transgene is expressed widely in neurons throughout the brain [188]; however, the PrP promoter is not permissive for expression in PCs but can be expressed in astrocytes [189]. Therefore, the transgene expression that was detected in the Purkinje layer is most likely located in the Bergmann glia. The PCs exhibited axon degeneration, demyelination, vacuolization, and a dark-cell degeneration phenotype that was also present in neurons in the granule layer. Large NIIs are visible in the granule neurons and contain the N-terminal part of hTBP with a polyQ expansion [190].

The SCA17 mouse model named L7-hTBP was generated using the shorter fragment of the *Pcp2* (L7) promoter with a full-length *TBP* cDNA containing 109 mixed CAA/CAG repeats [191]. The L7-hTBP mice express the transgene in the PCs of the cerebellum, but unlike with the regular *Pcp2* promoter, the expression is present in other brain regions including the brainstem, cerebral cortex, and hippocampus. Interestingly, the neuronal cell losses occur not only in the same brain regions but also in the striatum, pallidum, and hypothalamus. The animals do not exhibit reduced lifespans, but they show clear and early evidence of ataxia [191].

The first SCA17 knock-in model contains a chimeric mouse TBP exon 2 with 105 CAG repeats. However, the chimeric allele is inactive because of the floxed stop codon [192]. This kind of construct allows for endogenous (mouse promoter) and cell-specific expression of the mutant TBP that was demonstrated by neuronal expression using the Nestin-Cre mice [192]. The phenotype in the nestin-TBP knock-in mice is very mild, and the most distinctive phenotype, observed regardless of the ages of the mice, is a loss of nest-building behavior like that previously observed in an autistic mouse model [193]. Figure 3 contains a map of the neuropathology of the Nestin-Cre mice.

### DRPLA Mouse Models

The first mouse model of DRPLA (AT-FL-65Q) [194] is a PrP promoter-driven transgenic mouse line that expresses *ATN1* cDNA with either 24Q or 65Q. The AT-FL-65Q model develops a phenotype that consists of ataxia, tremors, aggression, and decreased breeding efficiency. The model has contributed important knowledge about the processing of mutant atrophin-1 protein in patients and mice. The transgenic atrophin-1 proteins with 65Q or 26Q undergo proteolytic cleavage, and the resulting 120-kDa fragments containing the mutant polyQ tracts accumulate in the nucleus, whereas the endogenous and 26Q atrophin-1 proteins are mostly cytosolic [194].

A similar but slightly more severe phenotype than that of the PrP mouse was observed for a pan-neuronal model of DRPLA using *ATN1* cDNA with 118 CAG repeats driven by a rat neuron-specific enolase (NSE) promoter [195].

DRPLA models with the entire *ATN1* gene and 76–78 CAG repeats were generated using the SuperCos 1 cosmid vector, but the expression of this transgene produces no obvious disease phenotype in these mice [196]. Later, intensive breeding of these mice [196] resulted in the creation of extremely expanded mosaic mice that have been bred to Q129 mice [197, 198]. Further related substrains with 113 and 96 CAG repeats were generated from Q129 strain [199].

The Q129 mouse phenotype is severe and highly complex; it features many aspects of the disease physiology and is similar to juvenile-onset DRPLA. In brief, the animals develop ataxia, myoclonic movements, and tactile-induced and spontaneous epilepsy [198]. These animals have severe brain atrophy manifesting as degeneration of dendrites and dendritic spines, presence of widespread NIIs and physiological changes in neurons, as recorded by electrophysiology [196]; however, a loss of neurons has not been observed [197] (for a map of neuropathology, see Fig. 3). Additional abnormalities in 129Q mice include polyuria and diabetes [198].

### SBMA Mouse Models

The initial attempts at generating an SBMA model produced mice with no phenotype [200, 201] or mice that lacked many of the features of the SBMA disease [202]. Neither the neurofilament light chain (NFL) promoter-driven model (NFL112) nor the PrP promoter-driven model (PrP112), which has a truncated form of the androgen receptor (AR), is able to reproduce the full SBMA disease phenotype.

#### SBMA Models Generated with AR Promoters

The AR-239Q mouse was the first mouse model that recapitulated many SBMA features [203]. These mice demonstrate that the 239 CAG repeats and the resulting protein composed exclusively of glutamines under the control of the human AR promoter are sufficient to evoke SBMA-like hind limb weakness and incoordination. The expression of the transgene and NIIs formation is evident in the brain, spinal cord, pituitary, lung, eye, and skin, all of which exhibit endogenous AR expression in mice [203].

The transgenic mouse lines have smaller brains, but there is no evidence of neuronal loss [203], which indicates that neuronal dysfunction is responsible for the pathogenesis observed in this mouse model.

A YAC construct containing a 450-kb genomic fragment that included the 100-kb human AR gene [201] with 100 CAG repeats was used to generate the SBMA AR100 model [204]. The model was able to reproduce the fact that the development of SBMA is sex-dependent. The AR100 males exhibit impaired mobility, hind limb atrophy, and occasional hind limb paralysis, while the female mice are only mildly affected. Anatomically, the muscle atrophy is likely caused by muscle denervation resulting from a very low number of motor neurons present in the lumbar spinal cord of the YAC animals [204, 205]. Males (but not females) have decreased expression of the VEGF164 protein isoform that is important for motor neuron survival [206]. Interestingly, the loss of endogenous AR expression aggravated the phenotype in the AR100 mice [207].

An SMBA knock-in mouse model was generated by replacing the 1,340-bp coding sequence of mouse AR exon 1 with a human exon 1 sequence containing 113 CAGs [208]. The AR113Q males are infertile and exhibit testicular atrophy, decreased body weight, and weak forelimbs [208, 209]. The muscle weakness and occurrence of early death in AR113Q are androgen-dependent, as confirmed by castration and testosterone supplementation experiments [209]. Moreover, the mice exhibit myopathic changes that are similar to those observed in SBMA patients. The myopathy is associated with the loss of chloride channel (CLCN1) protein expression in muscles, which can be rescued by castration [209]. Moreover, Ranganathan et al. [210] provided evidence for mitochondrial dysfunction in the AR113Q knock-in mouse model.

#### SBMA Models Generated with Nonendogenous Promoters

Multiple SBMA models were created using the full-length *AR* cDNA driven by a CMV (AR120 mouse) [211], β-actin (AR 97Q mouse) [212], and PrP promoter (112Q mouse) [213]. These models reproduce many features of SBMA, including the muscle weakness and atrophy, myelination defects and loss of motor neurons, and testicular pathology. As in previous SBMA models, testosterone deprivation by castration in the AR97Q model significantly improves the SBMA phenotype, demonstrating a direct correlation between the disease phenotype and androgen levels. Conversely, testosterone-treated female mice exhibit a phenotype that is similar to that of non-castrated male mice [212]. In addition, in the 112Q model, the males predominantly develop an overt phenotype and lose fertility, yet premature death is not present in this model. The mice lacked evidence of muscular atrophy or neuronal loss in the spinal cord while having the general polyQ phenotype [213–216].

#### Muscle-Specific SBMA Model

A mouse model, which supports a different, muscle-centric view of SBMA pathogenesis, overexpresses AR and contains 22 CAGs exclusively in skeletal muscle fibers under the control of the human skeletal α-actin promoter [217]. Endogenous mouse AR expression is blocked using the testicular feminization mouse (Tfm) genetic background. The resulting mouse strain—with exclusive and extremely high overexpression of AR in the muscles—has a phenotype that is similar to mouse models with much longer CAG repeats; these mice exhibit motor neuron pathology and deficits in muscle strength and motor function that are present in the males but not in the females. This high expression of the transgene in muscle may compensate for the lack of longer CAG repeats and therefore may evoke the disease phenotypes. The loss of motor function is androgen-dependent because female mice that are treated with testosterone develop the disease, while motor function returns in males after castration [218]. The marked loss of muscle strength and rapid loss of body weight (30 % over 9 days) in testosterone-treated females were quickly reversed when testosterone administration was stopped [218].

## The Data Table

### Comparison and Validation of Mouse Models

The approach of comparison and extrapolation of animal models to human diseases was historically proposed by Paul Willner [219]. He reasoned that the validation of animal models should be conducted according to general validation criteria that are already used in scientific research, namely, construct validity, face validity, and predictive validity [220]. These criteria were originally used for validating psychometric tests or psychiatric diagnoses and were proposed before identification of causative genes in polyQ disorders and also before the creation of the first polyQ mouse models.

#### Construct Validity

Construct validity should be assessed to determine whether a mouse model reproduces the molecular mechanisms that are responsible for the development of the human disease. The creation of polyQ mouse models with high construct validity is much easier than modeling complex pathology such as mood disorders, where mouse models have low construct validity [221]. On average, the construct validity of polyQ models is relatively high because these diseases have a well-defined etiology related to a specific type of mutation—expansion in known single genes. Although the creation of models of diseases related to single genes is relatively easy, several possible approaches exist. Two examples of such approaches are (1) creating a mouse model that contains a CAG expansion mutation in a mouse gene, such as HD knock-in models (references in “Knock-in HD models”), or (2) transferring the entire human mutant gene into the mouse genome, such as in the YAC128 or BACHD [27, 30] models. In the first approach, the mutation is deprived of its original context of the human gene, which may be very important for correct reproduction of the disease. In the second approach, two copies of the endogenous mouse gene are still present in the final mouse model. The additional presence of endogenous mouse genes may ameliorate the disease phenotype, as demonstrated in the YAC128 on the *Htt* knock-out background [222]. The wild-type huntingtin can also protect neurons from excitotoxicity [223]. The above examples illustrate that it may be difficult to evaluate which of these models has a higher degree of construct validity. The examples of models with relatively low construct validity are R6/2 and N171-82Q mice, which express only fragments of causative proteins and exhibit more features of general polyQ toxicity instead of matching the specific mechanisms of HD in patients.

A theoretical model for the creation of a polyQ mouse with very high construct validity should reflect the genetic features of human patients where one allele contains the copy of the gene with a pathogenic number of CAG repeats and a second allele that contains the same gene with a normal number of repeats. Preferably, it should be a targeted design with a complete human sequence driven by a native promoter that contains all of the appropriate regulatory elements. Unfortunately, it is quite likely that such a model would have a very mild disease phenotype also due to the presence of a CAG repeat number that is within the normal human range. Table 2 summarizes the factors that should be considered when evaluating construct validity. More discussion of construct validity parameters is found in “The promoter, the transgenic sequence, and the number of CAG/Q repeats” and “Mouse genetic background.”

**Table 2:** Construct validity in polyQ models

Animal model properties	Higher construct validity (arbitrary)	Lower construct validity (arbitrary)
Mutation	Pure CAG tract	Mixed CAA/CAG tract
Adult length of polyQ	As in human	Longer than in human
Juvenile length of polyQ	As in human	Longer than in human
Gene context	Human gene	Nonhuman gene
Translated sequence (exons)	Full gene models	Fragment models
5′UTR, 3′UTR, introns	Full gene models	Fragment and cDNA models;
Promoter	PolyQ promoter	Non-polyQ promoter
polyQ mRNA and protein	Level equal to level of non-mutant gene of a host	Much higher or much lower expression level
Number of transgene copies	1	2 or more
Single allele or biallelic	Two alleles with various CAG tract (like in patients)	Single allele
Model generation process	Targeted	Random
Host species	Primates > swine > mouse ≈ rat	Fruit fly > *C. elegans* > yeast

#### Face Validity

The face validity of an animal model reflects the phenotypic similarity between a mouse model and the associated human condition. It is obvious that a large number of mouse models of polyQ disorders exhibit neurological phenotypes and motor impairments that resemble those of patients suffering from polyQ diseases. However, many of the phenotypes seen in a particular mouse model are not equivalent to any symptom that occurs in human patients. For instance the R6/2 and N171-82Q models present many phenotypes that are not present in patients, such as an extremely short lifespan or widespread nonspecific neuropathology that includes defects in the cerebellum and other brain regions. In contrast, the R6/2 model also reveals HD-specific pathology in the striatum, cortex, and other tissues. Therefore, it is extremely difficult and probably unnecessary to assess the absolute face validity of a given mouse model, but it is important to identify the validity of certain aspects of a mouse phenotype that would be necessary for conducting particular research tasks. By collecting information about the phenotypes of polyQ mouse models, our data table provides a tool for assessing their face validity. Further discussion of face validity parameters can be found in “Phenotypes, disease onset, progression, and localization of neuropathology in the CNS and other tissues.”

#### Predictive Validity

The important reason for producing mouse models of human disorders is the evaluation of potential drug candidates. The predictive validity of a mouse model can in principle be defined as its ability to predict the molecular and symptomatic drug responses in a patient. Identifying the predictive validity of a mouse model requires data from testing various drugs in the mouse model and data about the clinical trials of treatments using the same substances in human patients. Our second data table and review (Part II) may be very useful for examining the predictive validity of polyQ models because we have collected most of the available data about therapeutic approaches in polyQ mouse models. Unfortunately, clinical trials for most of the drugs collected in the Part II data table have not been conducted so far, and the drugs that have been evaluated, such as CoQ10, ethyl-eicosapentaenoate (ethyl-EPA), tetrabenazine, memantine, phenylbutyrate, dimebolin hydrochloride, and minocycline, either were unsuccessful or produced limited therapeutic benefits [224, 225]. Moreover, all of these substances had been preclinically tested on R6/2 model animals and had shown improvement in the disease phenotype (see the Part II data table). For the reasons described above, the predictive validities of polyQ mouse models are still unknown. Regardless of their low construct validity, low face validity, and unknown predictive validity, the R6/2 and N171-82Q models are the most extensively used models in research on both the HD pathogenesis and therapy.

### The Promoter, the Transgenic Sequence, and the Number of CAG/Q Repeats

The promoter, the transgenic sequence, and the number of CAG/Q repeats (which are also listed in the data table) influence the effectiveness of reproducing the polyQ disease phenotype in mice. They also provide information about the properties of the transgene cassette and on the resulting expression pattern in mice. This expression pattern is shaped by the transgene promoter, which can drive expression in all cells (pan-cellular promoters), all neuronal cells (pan-neuronal promoters), or specific cell types, such as PCs. To model the disease as accurately as possible, researchers also use native human and mouse promoters for generating knock-in and YAC models. Our data table lists three principal promoters that have been used to create the polyQ models: the PrP promoter, which was used in 21 mouse models; the huntingtin (mouse and human) promoter, used in 19 models; and the *Pcp2* promoter, used for generating 12 models. Together, these promoters were used to generate over half of all polyQ models (Supp. Fig. 3).

#### The Impact of Promoters and Transgene Sequence in polyQ Mouse Models

In addition to the PrP promoter, the pan-neuronal and pan-cellular group of promoters includes the NSE, NFL, CMV, and PDGF-B promoters. The PrP promoter has been widely used to create polyQ models of HD, DRPLA, SCA1, SCA3, SCA7, SCA17, and SBMA. The natural prion protein is expressed in mice ubiquitously throughout the brain; however, its expression in the striatum (caudate and putamen) is quite weak (see, for example, the Allen mouse brain atlas). Moreover, the engineered version of this promoter does not drive expression in PCs, and its expression in the striatum appears to be weak [226, 227]. The expression profile of this promoter has been examined in the mouse brain using PrP-LacZ expression. The authors did not detect this promoter activity in MSNs in the striatum and only found expression in interneurons [228]. We can corroborate this finding, as in our hands, the transgenic protein levels and NIIs in the striatum of the PrP-driven N171-82Q model are barely detectable, whereas these inclusions are frequent in the cerebral cortex and hippocampus [169]. The causative proteins huntingtin, ataxins 1, 2, 6, and 7, and atrophin-1 are all naturally expressed in the striatum and PCs.

Moreover, although the PrP-based models express different proteins with polyQ tracts, the resulting mice present a very similar set of phenotypes in which the severity of the phenotype and shortened lifespan are characteristic hallmarks. For example, the PrP promoter has also been used to create the SBMA model (PrP112) [202], which exhibits a very strong phenotype that resembles the phenotype observed in N171-82Q mice but has an even shorter lifespan. The PrP112 model reveals more severe general impairment and lacks slowly progressing SBMA phenotypes, such as weak hind limbs and weak gait. In general, many PrP-based models with long pathogenic CAG tracts have a dramatically decreased lifespan and severe impairments. It is likely that the cellular effects of the polyQ tracts and similar expression patterns of the transgenes in various PrP mice strongly contribute to the disease phenotype, whereas differences in protein context have a weaker influence on the disease. A somewhat stronger manifestation of protein context is present in some PrP models, such as models of SCA3 and SCA7. Ataxin-3 is expressed ubiquitously in the mouse brain, including the cell soma of PCs. Interestingly, although the PrP promoter is not expressed in PCs, the transgenic models that express full-length human mutant ataxin-3 under the PrP promoter exhibit a loss of the dendritic tree in the Purkinje cell layer. However, in transgenic animals, PrP/expanded ataxin-3 is expressed in similar brain regions to AR in the SBMA PrP112 model and the huntingtin fragment in the N171-82Q model, yet the pathogenic effects are clearly distinct, indicating an influence of ataxin-3 protein context. The influence of AR protein context can be observed in the SBMA 112Q model, which is the full-length AR PrP-driven model [213]. The influence of protein context is also evident in the PrP/SCA7 model with 92Q, in which the mice develop a loss of vision that is similar to that of human patients. However, this is accompanied by a very severe phenotype and premature death, which are much like the PrP112/SBMA and N171 models. We conclude that the PrP promoter has the potential to reproduce some of the protein-specific symptoms of human polyQ diseases. Unfortunately, it also promotes many severe and nonspecific phenotypes that may be attributed to general polyQ effects; therefore, it is uncertain which kind of disease the resulting mice really mimic.

The cell-specific group of polyQ transgenic constructs uses the *Pcp2* (L7) promoter for PCs, the dopamine- and cAMP-regulated neuronal phosphoprotein (DARPP32) promoter for MSNs in the striatum, the GFAP (Gfa2) promoter for various populations of astrocytes, including Bergmann glia, the nestin and Emx1 neuronal promoters for Cre-induced animals, and the human skeletal α-actin promoter to drive expression in muscle cells. Using the cell-specific promoters to generate animal models has revealed interesting information regarding the cellular pathogenesis of polyQ diseases.

Both ataxin-1 and ataxin-2 are prominently expressed in PCs, and therefore, the *Pcp2* promoter is a good first choice for several SCA1 (and one SCA2) mouse models that show degeneration of PCs as the most striking event in SCA pathology. In patients, the degeneration of PCs is present, but the most prominent disease marker, NIIs, are barely detectable in these cells. We also know from the conditional SCA1/Prp-tTA and SCA3 PrP/MJD77 mouse models that PCs degenerate without transgene expression because the PrP promoter does not drive expression in PCs [229, 230]. This finding suggests that the damage of PCs in SCA may be non-cell-autonomous and may be the result of some primary pathogenic condition instead. These conditions may be the result of the pathologic influence of other cells or lack of the physiologic influence of these cells because of their damage or loss. This model of nonautonomous degeneration was recently proposed for other polyQ diseases. It has been demonstrated (using the GFAP promoter) that Bergmann glia may play an important role in the pathogenesis of SCA7 [177]. In addition, the GFAP promoter and mutant HTT fragment were recently expressed in transgenic mice, which resulted in an HD-like phenotype [231] in addition to exacerbating HD neuropathology in the N171-82Q model [232]. These mice have provided evidence for the involvement of astrocytes and the strong contribution of aberrant glutamate uptake in SCA7 and HD. This finding suggests that excitotoxic death of neurons in cerebral cortex may lead to loss of connectivity to striatum and thus may play role in evoking HD. This hypothesis is in agreement with results that were obtained from mouse models that restricted the expression of the HTT transgene to the striatum and MSNs with either a conditional model or the DARPP32 promoter [23, 36, 233]. In those models, the striatum and MSNs are the only affected populations in the mouse brain; however, this does not produce a drastic phenotype compared to the pan-neuronal models of HD.

Recently, it has been confirmed that in addition to glia and neurons also endothelial cells in the brain and the abnormal vasculature investigated in YAC128 model may participate in pathogenesis of HD [234]. Another example of redefining the pathogenesis of polyQ disorders is SBMA. It has been demonstrated that the mouse model that uses the HSA promoter to express the transgene in muscle cells can elicit SBMA-like phenotypes in mice [217]. This finding suggests that the degeneration of motor neurons in SBMA may be secondary (non-autonomous pathogenesis) and dependent on primary muscle degeneration. The SBMA phenotype in mice also appears to rely heavily on both the mutant polyQ tract and its expression pattern because mice that have an AR promoter driving the expression of a protein composed exclusively of 239 glutamines show SBMA-like phenotypes [203].

The mouse and human versions of native promoters for polyQ genes have been used in knock-in models and in transgenic R6/2, YAC, BAC, and Cosmid models. Generally, the knock-in and YAC models of polyQ disorders produce no phenotype, a weak phenotype or develop the disease much more slowly than the simple transgenic models and this applies to HD models in particular. The random transgenic models often contain many transgene copies that result in very strong transgene expression. However, the slow phenotype cannot simply be explained by the number of integrated transgenes because the phenotype severity does not always increase with an increase in the number of transgene copies. For example, in the R6 lines, although the R6/5 has five transgene copies and the R6/1 and R6/2 lines have only one copy each, the phenotype severity does not mirror this difference [19]. Similar observations were also made among the SCA1 B0X lines [14]. Nevertheless, mice that have been bred to homozygosity, like HD knock-ins or SCA6_84Q [17], are more affected. Their severe phenotype may result from either increased mutant protein expression from two alleles or the lack of mitigating activity of a wild-type allele, which was investigated in a study of YAC128 mice on an Htt-knock-out background. In YACs, BACs, and knock-in models, the protein expression level is usually lower than that of the endogenous allele. Moreover, these discrepancies exist despite high levels of mRNA expression. For example, in the YAC128 model, which has transgene mRNA expression several orders of magnitude higher than WT mRNA expression, the level of transgene protein expression is only 75 % of the WT level [30]. Similar puzzling discrepancies concerning the expression of the *Htt* gene were identified in the Hdh(CAG)150 knock-in mouse model, in which the expression of mutant protein is variable and lower compared to that of the WT allele [235]. It has also been postulated that the length of the transgene protein contributes to the severity of the disease in HD models. Models with a short mutant protein, such as the R6/2 and N171-82Q models, show phenotypes much earlier because of the skipping of a putative protein-processing step that would otherwise delay the onset of the disease in animals that have longer proteins. Moreover, the N-terminal 586 caspase-6 fragment seems to be just the intermediate step in HD patogenesis since the 586-82Q models [236, 237] exhibit milder phenotypes than R6/2 and N171-82Q. The neurons from 586-82Q models contain predominantly cytoplasmic agregates and such localization may suggest that the pathogenic accumulation of HTT fragments in nucleus is delayed. However, the transgene length hypothesis is not supported by the Shortstop model, which contains a short protein but does not show an HD-like phenotype [20]. Recent work concludes that the lack of such a phenotype cannot be completely explained by the properties of the mutant N118 htt protein fragment because the mice containing this protein under the PrP promoter had pronounced phenotypes [24]. The clear differences between the Shortstop model and the R6/2 or N118 models are the 5′ sequences and promoter sequences that are present in these models. The Shortstop model contains large flanking sequences from the 5′ end, along with the entire *HTT* promoter; together, these comprise 24 kb of upstream human sequence, whereas the R6/2 contains only 1 kb of the *HTT* promoter. YAC and knock-in models also contain entire *HTT* and *Htt* genes with flanking sequences and therefore preserve the endogenous signals and gene vicinity that influence the regulation of *HTT* and *Htt* gene expression. Interestingly, it has been postulated that nongenetic factors influence the regulation of the expression of *HTT* and *Htt* [235]. These nongenetic factors, such as chromatin remodeling, can modulate the expression of *HTT* and *Htt* genes by driving the expression in defined cell types and tissues such as the brain and the testes [235]. Perhaps, the deletion of the entire 3′ sequence in the Shortstop model may cause gene regulation via epigenetic chromatin remodeling or antisense *HTT* transcripts [238] that results in the selective silencing of the shortstop protein in certain neuronal cell types that are particularly involved in pathogenesis of HD. According to such hypothesis, the 5′ untranslated region and the promoter of the *HTT* gene would be responsible for the severity of disease in mouse models, and probably also in patients.

#### The Impact of CAG Repeat/polyQ Tract

Unlike patients who suffer from polyQ disorders, the mouse models are not very sensitive to the number of CAG repeats in the transgenes. To evoke a phenotype in mice, the average number of repeats in the transgene needs to be higher than in humans. Mice have a relatively short lifespan, and it is likely that the accumulation of the pathogenic events is sometimes insufficient to evoke the phenotype. Moreover, the positive correlation between the number of repeats and disease severity that is present in human patients is not as apparent in mice. This correlation can be observed in HD YAC, HD knock-ins, and SCA1 knock-ins, which have similar model variants with increasing numbers of CAG repeats. Unfortunately, this positive correlation cannot be confirmed for transgenic, high copy integration mouse models because of expression levels that “balance” the CAG counts. The phenotypes in transgenic mice are strongly influenced by expression level. For example, two copies of a transgene may have a dramatic effect, whereas one copy may leave the mouse healthy, as occurs in some HD knock-ins, Sca3Q71-b, and PrP/MJD77 models. In addition, the increased number of repeats may sometimes ameliorate the disease phenotype rather than accelerating it, as in the case of R6/2 mice variants with very long CAG tracts [38, 39]. Moreover, it is quite puzzling that the expression of the protein containing expanded polyQ tract does not always elicit a diseased phenotype. For instance, the Shortstop HD animals contain both inclusions and mutant protein expression, but show no signs of disease [20]. Similarly, full-length ataxin-3 expression in PCs produces no phenotype, but a fragment of ataxin-3 does produce a phenotype [154]. Moreover, HD models that express either the caspase-site-mutated [26] or phosphomimetic [239] versions of huntingtin are rescued from the disease despite having an expanded polyQ tract. Similarly, a recent YAC128 model that completely lacks caspase-2 expression does not show the behavioral changes that are typical in HD models despite exhibiting neurodegenerative alterations such as reduction in striatal volume [240]. Conversely, the polyQ tract is not always needed to evoke the disease, as in the SCA model, which has a substitution of Ser776 with a phosphomimicking Asp residue [138].

In summary, properties of the transgenic cassette and the promoter type that is used in a transgenic construct have a prominent impact on the disease phenotype. Additional features that were not included in the transgenic properties section of our data table are the transgene’s mRNA and protein levels and the number of transgene copies that are integrated into the mouse model. This omission is mainly due to a lack of data. The few studies that report precise values of transgene expression levels in relation to endogenous levels would not significantly enrich the data table. However, gathering such data from all of the polyQ models would be useful for standardizing the phenotypes and investigating the outcomes of therapeutic approaches.

### Mouse Genetic Background

The careful selection of genetic background for maintaining transgenic mice is important for the reliable characterization and comparison of mouse models because various strains exhibit differences in physiological, motor, and cognitive traits [97, 241–245]. The genetic background can influence a number of features, including breeding qualities, behavioral responses to changes in environmental conditions [246–248], and testing paradigms [249–251], all of which may generate lab-to-lab variability in phenotypic outcomes [252]. Thus, the performance of the background strain in a particular test can influence the performance of a transgenic model maintained on this background. For example, some popular strains (C3H, CBA/J, FVB/NJ, and SJL/J) are homozygous for the retinal degeneration allele rd1, which results in blindness [253] at a young age. These strains are not optimal backgrounds for modeling diseases with visual impairment, such as SCA7, where testing of visual performance is essential. Visual impairment may influence the outcome of spatial learning in the Morris water maze [254], or it may influence strategy-shifting tasks that are based on visual discrimination. Similarly, the hearing loss in 129/SvJ, DBA/2J or older C57BL/6J [255] strains may influence performance on acoustic startle and PPI tests [256].

Our data table includes information about the genetic background of the models, and we have presented it according to the Jackson Laboratory nomenclature for mouse strains. The models were often characterized based on different backgrounds and stages of backcrossing.

Most of the information about the influence of genetic background in polyQ diseases is again related to HD mouse models. The R6/2 model is generally bred using the F1 hybrid of the C57BL/6J strain with either UK-bred CBA or CBA/J animals [45]. The two substrains are significantly different because they reject skin grafts between each other [257], and, unlike the UK substrain, the CBA/J animals carry a homozygous rd1 allele, thereby contributing a 25 % chance of blindness to the R6/2. UK- and US-bred R6/2 animals show differences in acoustic startle performance, PPI performance and body weight loss [42, 45]. However, it is presently uncertain whether the difference in genetic background contributes to phenotype discrepancies between UK- and US-bred mice because the existing data either are based on separate studies with different methodologies [38, 45, 258] or compare mice with different lengths of the polyQ tract [45]. Therefore, a more systematic comparison among R6/2 variants with shorter and longer CAG tracts on hybrid and pure C57BL/6J backgrounds is needed.

Like the R6/2 mice, R6/1 mice show differences in acoustic startle and PPI performance, body weight loss, and locomotor activity depending on their genetic background [41, 259–261]. This issue also requires further clarification.

In the case of the YAC 128 model, different genetic backgrounds have been compared in cross-sectional studies [45, 93]. The most popular YAC128/FVB/N combination has a more pronounced phenotype than the less affected YAC128 on 129/SvJ or C57BL/6J backgrounds. The YAC128/129/SvJ strain also lacks the hypoactivity [93] phenotype, which is probably related to increased anxiety, pronounced hypoactivity, and lack of motivation in the 129/SvJ strain [245].

In the HdhQ111 model, intergenerational and somatic stability of CAG expansions are affected by the genetic background, as is the formation of polyQ NIIs. HdhQ111 mice on a C57BL/6NCrl congenic background develop the most pronounced CAG tract instability and the greatest number of NIIs, whereas the CAG tracts were rather stable, and few NIIs were observed in HdhQ111 on a 129/SvCrl background. The phenotype on an FVB/NCrl background is placed in the middle of C57BL/6NCrl and 129/SvCrl strains [262].

Similar to HdhQ111 model, greater CAG tract instability was observed in the SCA3 CMVMJD94 mouse model on a C57BL/6J background than on an FVB/N congenic background [168]. Another study of SCA1 78Q mice revealed that backcrossing from a mixed C57BL/6J;129/SvEv background to a pure C57BL/6J background is able to rescue the rotarod deficit [145]. The SCA1 B05 model on the mixed FVB/N;129/SvEv background had a slower progression of phenotype development compared to mice that were backcrossed to a FVB/N [145] background. Like in Sca1 78Q mice, backcrossing the SCA7 B7E2 mice from a (C57BL/6J × SJL)F1 background to a pure C57Bl/6J background ameliorated the phenotype [171].

### Phenotypes, Disease Onset, Progression, and Localization of Neuropathology in the CNS and Other Tissues

In our data table of mouse models and phenotypes, we collected 41 groups of “phenotypes” (Table 3), including almost 200 “detailed phenotypes” in 107 mouse models and variants that model nine polyQ diseases. Both a detailed description of a system for describing the phenotypes in our data table and the data table itself are available online as Supplementary material.

**Table 3:** The 41 groups of “phenotypes” identified in polyQ models belonging to “general phenotypes” categories [neuropathological (N), cognitive (C), motor (M) and other (O)]: the number indicates the amount of records that are present for every “phenotype” in the data table

Neuropathological (N)		Other (O)			
polyQ protein aggregates	549	Lifespan	69	Hormonal dysregulation	13
Neuronal morphology alterations	145	Organ pathology	54	SHIRPA change	6
Neuronal physiology alterations	138	Body weight change	54	Body temperature alteration	4
Brain morphology alteration	88	polyQ protein aggregates	51	Circadian rhythm alteration	3
Neuronal loss	62	Muscle abnormalities	42	Abnormal urination	2
Glial abnormalities	55	Posture abnormalities	32	Hearing impairment	2
Retinopathy	16	Fertility	19	Diabetes insipidus	1
Abnormal myelination	10	Adipose tissue dysfunction	15	Narcolepsy	1
Sensorimotor gating impairment	10	Metabolism alteration	14	Visual impairment	1
Neurogenesis impairment	8	Diabetes mellitus	13		
Autonomous nervous system (ANS) dysfunction	1				
Cognitive (C)		Motor (M)			
Learning and memory impairment	42	Balance and coordination alterations	111	Tremor	25
Affective function alteration	23	Locomotor impairment	97	Abnormal sensorimotor response	21
		Gait alteration	52	Seizures	11
		Clasping behavior	37	Dyskinesia	7
		Muscle strength impairment	29		

Table 3 summarizes the 41 phenotypes that belong to motor (M), neuropathological (N), cognitive (C), and other (O) “general phenotypes”. A large portion of these phenotypes (approximately 32 %) are polyQ protein aggregates. Because the mice have aggregates in multiple tissues, nuclear inclusions are listed in the data table repeatedly for different tissues and brain regions. The detailed phenotypes in the polyQ protein aggregates group are aggregates, aggresomes, amyloid-like inclusions, cytoplasmic aggregation foci (AF), diffuse cytoplasmic staining, diffuse nuclear staining, extranuclear aggregates, nuclear inclusions, and nuclear microaggregates. Neuronal morphology alterations occur frequently in the mouse models and contain detailed phenotypes, including abnormal cell morphology, cell size decrease, dark cell degeneration, and dendritic degeneration. Locomotor impairment was measured as the frequency of grooming, rearing, and motor and exploratory activity, and the balance and coordination alterations category included observations of rotarod or beam walk impairment.

We investigated whether we could identify groups of detailed phenotypes that are universal and phenotypes that are unique to mouse models of different disorders. Using the data in our data table and pivot table tools in Excel, we pooled detailed phenotypes from all mouse models of a particular disease and investigated the overall similarities and differences among phenotypes associated with diseases that were reproduced in mouse models.

We identified 21 detailed phenotypes that were present in mouse models that represented at least 6 diseases and 17 detailed phenotypes that are common to mouse models that represent 4 or 5 diseases (Table 4). We identified a third group of 33 detailed phenotypes that are present in mouse models that represent 2 or 3 polyQ diseases. Finally, 124 different detailed phenotypes are unique to a single polyQ disease model or to a single disease. The phenotype that is present in the mouse models of all diseases is rotarod impairment. The cell size decrease, clasping behavior, and gait abnormality phenotypes are present in all of the mouse models except models of SCA6.

**Table 4:** The phenotypes overlapping across mouse models and diseases

	Detailed phenotypes	Diseases								
Common for 6 or more diseases	Rotarod impairment	DRPLA	HD	SBMA	SCA1	SCA2	SCA3	SCA6	SCA7	SCA17
	Abnormal gait	DRPLA	HD	SBMA	SCA1	SCA2	SCA3		SCA7	SCA17
	Cell size decrease	DRPLA	HD	SBMA	SCA1	SCA2	SCA3		SCA7	SCA17
	Clasping	DRPLA	HD	SBMA	SCA1	SCA2	SCA3		SCA7	SCA17
	Ataxia	DRPLA	HD	SBMA	SCA1		SCA3		SCA7	SCA17
	Body weight loss	DRPLA	HD	SBMA	SCA1		SCA3		SCA7	SCA17
	Cell loss		HD	SBMA	SCA1	SCA2	SCA3		SCA7	SCA17
	Decreased lifespan	DRPLA	HD	SBMA	SCA1		SCA3		SCA7	SCA17
	Dendritic degeneration	DRPLA	HD		SCA1	SCA2	SCA3		SCA7	SCA17
	Diffused nuclear staining	DRPLA	HD	SBMA	SCA1		SCA3		SCA7	SCA17
	Electrophysiology alteration	DRPLA	HD	SBMA	SCA1		SCA3	SCA6	SCA7	
	Gene expression alteration	DRPLA	HD	SBMA	SCA1		SCA3		SCA7	SCA17
	Motor and exploratory activity decrease		HD	SBMA	SCA1		SCA3	SCA6	SCA7	SCA17
	Nuclear inclusions	DRPLA	HD	SBMA	SCA1		SCA3		SCA7	SCA17
	Abnormal cell morphology	DRPLA	HD	SBMA	SCA1		SCA3		SCA7	
	Brain volume decrease	DRPLA	HD	SBMA			SCA3		SCA7	SCA17
	Brain weight loss	DRPLA	HD	SBMA	SCA1		SCA3			SCA17
	Diffused cytoplasmic staining		HD	SBMA	SCA1	SCA2	SCA3		SCA7	
	Kyphosis		HD	SBMA	SCA1		SCA3		SCA7	SCA17
	Seizures/evoked seizures	DRPLA	HD	SBMA			SCA3		SCA7	SCA17
	Tremor	DRPLA	HD	SBMA			SCA3		SCA7	SCA17
Common for 4 or 5 diseases	Apoptosis increase		HD	SBMA			SCA3		SCA7	SCA17
	Dark cell degeneration		HD	SBMA			SCA3		SCA7	SCA17
	Loss of calbindin				SCA1	SCA2	SCA3		SCA7	SCA17
	Reactive gliosis		HD		SCA1		SCA3		SCA7	SCA17
	Reduced fertility	DRPLA	HD	SBMA			SCA3		SCA7	
	Axonal degeneration	DRPLA	HD	SBMA						SCA17
	Demyelination/thin myelin		HD	SBMA			SCA3			SCA17
	General incoordination	DRPLA	HD		SCA1				SCA7	
	Grooming activity decrease		HD				SCA3	SCA6		SCA17
	Hindlimb dragging		HD	SBMA		SCA2	SCA3			
	Hypothalamic–pituitary–adrenal axis alteration	DRPLA	HD	SBMA			SCA3			
	Infertility		HD	SBMA			SCA3		SCA7	
	Layer thickness decrease (cerebellum)				SCA1		SCA3		SCA7	SCA17
	Motor and exploratory activity increase	DRPLA	HD		SCA1		SCA3			
	Muscle atrophy		HD	SBMA	SCA1		SCA3			
	Nuclear microaggregates		HD	SBMA					SCA7	SCA17
	Simple motor learning deficit		HD		SCA1		SCA3		SCA7	

The 21 “detailed phenotypes” (light gray) that are present in mouse models representing at least 6 diseases and 17 “detailed phenotypes” (dark gray) that are common for mouse models representing four or five diseases. For the number of “detailed phenotypes” in mouse models, see also Supp. Fig. 2

Ataxia, body weight loss, decreased lifespan, diffused nuclear staining, gene expression alteration, and nuclear inclusions are detected in all mouse models except SCA2 and SCA6. In contrast, diffused cytoplasmic staining or extranuclear inclusions phenotypes are found in both the SCA2 and SCA6 models. The dendritic degeneration phenotype was not reported for SBMA mouse models, but other phenotypes, such as cell loss and dark cell degeneration, were present in SBMA models, indicating that also dendritic degeneration is part of the pathogenesis of SBMA. The cell loss phenotype was not present for the DRPLA and SCA6 models, indicating that these mouse models involve cell dysfunction without the loss of neurons. Tremor (which is a symptom that originates in the peripheral nervous system) was not present for SCA1, SCA2, or SCA6 mice, which is surprising for SCA1 models because of the involvement of the PNS. Although normal lifespan is not a phenotype per se, it was listed in the data table for many mouse models that exhibit neurological phenotypes. Moreover, a number of mouse models present only mild disease phenotypes and have only a slightly affected lifespan.

Unique phenotypes account for more than half of the phenotypes that have been identified in polyQ mice. For instance, the SBMA models display muscle pathology, while some cognitive phenotypes are specific to HD models.

In summary, many of the phenotypes that are associated with a given polyQ disease are common to mouse models of different polyQ diseases, which may indicate that the polyQ diseases are really similar disorders with many common aspects of pathogenesis. In addition, the models also reproduce many unique features of human polyQ diseases. The existing similarities between disease models may also indicate that we need more sophisticated and accurate mouse models to study polyQ disorders because some common phenotypes between current polyQ disease models may result from the way in which the transgenic mice were created (random integration, genetic background, and regulatory sequences).

#### Defining the Onset and Progression of the Disease in PolyQ Mouse Models

Our data table also lists “age of earliest detection (age I, weeks)” and “age of latest detection (age II, weeks)”, which indicate the ages at which an alteration was documented for the first or last time, respectively. The research works that were the sources of data for these columns were preselected; in the case of the column labeled age of earliest detection, the research works that reported the youngest mouse with a documented alteration in phenotypes that have a simple non-statistical nature, such as nuclear inclusions (present/not present), were selected. If the alterations were subjected to statistical analyses (for example, as in the case of behavioral tests, such as the rotarod, open field assays, cognitive tests, and brain volume assays), then the data were based on the youngest mice that had a documented, statistically significant alteration. The selection of data for the age of latest detection column was based on reports of the ages of the oldest mice with documented alterations that were statistically significant in the case of experiments that involved statistical analyses. One caveat is that the phenotypes obviously do not have endpoints, as a measure of the time of the last investigation might suggest, but the phenotypes may undergo transitions, such as a transition from hyper- to hypoactivity. Other example of such transition is the conversion of mutant polyQ proteins from diffused to aggregated forms. Obviously, this column contains fewer data, and the data are less reliable because researchers do not always follow the development of all phenotypes until very late ages in mice. The major reason for this lack of data is the fact that the model animals should not be tested too frequently because testing per se influences the phenotype and therefore precludes the accurate assessment of natural disease progression.

Based on the data collected in our data table, we have investigated the disease onset and progression in mouse models. The definition of disease onset in mouse models can be expressed as the single age of an animal when the first change in phenotype was detected. This definition has several drawbacks. First, this definition assumes that a deficit or phenotype begins when it is first detected or investigated, not when it actually appears in the animal. At present, researchers cannot avoid making this assumption; however, they must always realize that the age of disease onset in mice is an approximation that can always be refined by more intensive research and the use of more sensitive methods. The second disadvantage is that this definition limits the description of onset to a single time point at which either a limited number of phenotypes or only one phenotype starts to occur. However, disease onset and pathogenesis result from several events that initiate the development of phenotype. Therefore, the age of disease onset in mice can be investigated as a time frame that encompasses the earliest onsets of phenotypes from three major groups of phenotypes, including motor, neuropathological, and cognitive phenotypes. These data are collected in our data table. To investigate this time frame in different models, we have prepared Table 5, which lists the ages of onset for motor, neuropathological, and cognitive phenotypes.

**Table 5:** Characteristics of representative polyQ mouse models

	Model name	Motor		Neuropathology		Cognitive		Inclusions	Lifespan	AD50
		Age	Test (phenotype)	Age	Phenotype	Age	Test (phenotype)	Age		
HD	R6/2	4	Acoustic startle PPI test (acoustic startle response reduction); open field (motor and exploratory activity decrease, rearing rate decrease); rearing climbing test (climbing ability decrease); rotarod (impairment); tail suspension test (dystonic movements)	2	Gene expression alteration; postnatal myelination deficit	3.5	Morris water maze (spatial learning and memory deficit)	1	15	8
	R6/1	4	Open field (motor and exploratory activity increase)	5	Neurotransmitter receptors level alteration	7	Open field habituation (habituation deficit)	9	32–40	16
	N171-82Q	8	Open field (rearing rate decrease)	6	Brain volume decrease	13	Rotarod (simple motor learning deficit)	4	28	13
	BACHD	4	Open field (rearing rate decrease); rearing climbing test (climbing ability decrease); rotarod (impairment)	2	Demyelination/thin myelin; postnatal myelination deficit	12	Light/dark choice test (anxiety increase)	52	Normal	17
	YAC128	9	Open field (motor and exploratory activity increase)	5	Electrophysiology alteration	9	Rotarod (simple motor learning deficit)	65	Slightly decreased	35
	CAG140	4.5	Open field (motor and exploratory activity increase, rearing rate decrease)	8	Brain metabolites level alteration	6.5	Fear conditioning test, light/dark choice test (anxiety increase)	9	Normal	30
	Hdh(CAG)150	20	Rotarod (improvement); tail suspension test (clasping)	26	Brain volume decrease; cell loss; gene expression alteration; prepulse inhibition deficit	17	Morris water maze (spatial learning and memory deficit)	37	Normal	43
SCA1	B05	5	Bar cross apparatus, open field (motor and exploratory activity increase); rotarod (impairment)	4	Abnormal cell morphology	5	Rotarod (simple motor learning deficit)	6	Normal	10
	SCA1 154Q	5	Rotarod (impairment)	16	Brain weight loss	5	Rotarod (simple motor learning deficit)	6	45	19.5
SCA3	Q71-B	8	Grip strength meter (grip strength decrease); rotarod (impairment)	–	–	–	–	13	36	17
	MJD84.2	4	Observation (abnormal gait)	6	Electrophysiology alteration	–	–	+^a^	Normal	41
SCA7	PrP-SCA7-92Q	8	Rotarod (impairment)	7	Photoreceptors dysfunction	–	–	4	34	13
	SCA7 266Q	5	Observation (muscle weakness, tremor); rotarod (impairment)	4	Gene expression alteration	–	–	5	19	8
SCA17	L7-hTBP	9	Observation (ataxia), rotarod (impairment)	4	Cell loss; cell size decrease; dendritic degeneration; loss of calbindin	–	–	–	Normal	13
DRPLA	Q129	3	Observation (ataxia, seizures)	4	Brain volume decrease; brain weight loss; electrophysiology alteration; gene expression alteration	–	–	9	16	8
SBMA	112Q	6	Tail suspension test (clasping)	61	Cell loss	26	Elevated plus/zero maze (anxiety decrease)	6	Normal	30
	AR113Q	8	Grip strength meter (grip strength decrease)	4	Gene expression alteration	–	–	104	20/110a	13

^a^For further details, see “The data table”

**Fig. 4:**
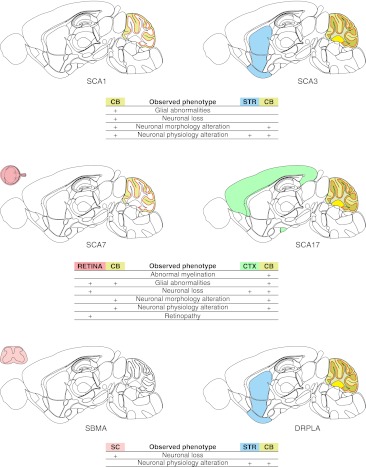
Diagrams of brain, spinal cord, and retina neuropathology in polyQ models that use the PrP promoters. As in previous figures, the phenotypes and brain regions were marked with colors on a schematic sagittal section of the mouse brain. Because multiple PrP polyQ models were available for some diseases, the data from multiple models for each disease were pooled to increase the total information content. The PrP promoter produces a pattern of neuropathology that is different from that produced in models that use native promoters. The PrP promoter tends to produce neuropathological outcomes in the cerebral cortex (*CTX*), cerebellum (*CB*), and striatum (*STR*), but, unlike the native promoters (Fig. 3), the PrP promoter does not tend to produce pathology in the hippocampus (*HP*)

**Fig. 5:**
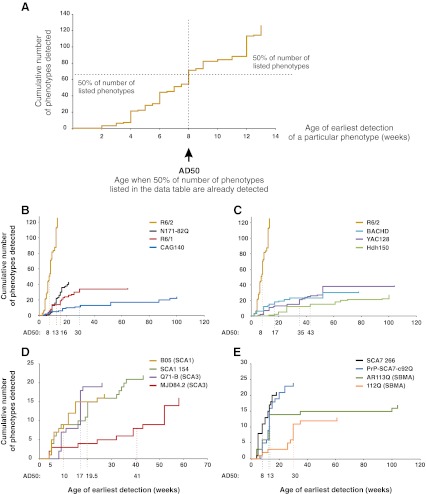
Graphs of disease progression in polyQ mouse models and the AD50 parameter. We have constructed a normalized value that describes the progression of the disease in a mouse model and that allows for comparison between mouse models. This is the AD50 (age at 50 % detected phenotypes), which represents the median of the values from the “age at earliest detection” column of the data table for an analyzed mouse model. The AD50 is the age at which 50 % of the total number of phenotypes for a particular mouse model has been detected and reflects the dynamics of disease progression in that model. The 50 % of number of phenotypes is a normalizing value for the different numbers of phenotypes described in various polyQ mouse models. **a** An example graph that shows how the graphs of disease progression are constructed for various models and where the AD50 is located on the graph for the models. **b**–**e** The graphs of progression of the disease for various models representing various polyQ diseases. The R6/2 contains 134 “detailed phenotypes” in the data table (after excluding polyQ aggregates and some phenotypes without an “age of earliest detection”). Thus, 50 % of the number of phenotypes equals 67 for the R6/2 model and 19 for the YAC128 model. The AD50 for the R6/2 model is 8 weeks and that for the YAC128 model is 35 weeks. Thus, the AD50 values reflect the different speed of disease progression in each model. The analysis excludes the “polyQ protein aggregates” phenotypes for reasons that are described in the main text

We also asked whether it would be possible to use our data table to construct a normalized value that would both describe the progression of the disease in a given mouse model and allow for comparison between mouse models. Every model in the data table contains a set of phenotypes, the majority of which have a specified age of earliest detection. For instance, the R6/2 model on the hybrid background contains 164 phenotypes, and the age of earliest detection is specified for 158 of them. The age at which 50 % of these phenotypes have been detected in a particular model reflects the dynamics of disease progression in that model (Fig. 5). We have excluded the “polyQ protein aggregates” phenotype from this analysis for reasons described below. Thus, 50 % of the number of specified phenotypes would be 67 for the R6/2 model and 19 for the YAC128 (FVB/N) model. Therefore, the 50 % of phenotypes can be used as a normalizing value for the different number of phenotypes described in various polyQ mouse models (Fig. 4). Thus, we propose a parameter AD50 (age at 50 % detected phenotypes), which is defined as the age at which 50 % of the number of phenotypes for a particular mouse model have been detected. The AD50 is the median of the values collected in the column age of earliest detection for each analyzed mouse model. The AD50 for the R6/2 model is 8 weeks, and the AD50 for the YAC128 model is 35 weeks reflecting the different speed of disease progression in the two models. The above analysis was based on a number of assumptions. The first is that the disease in a mouse model is progressive and the number of phenotypes gradually increases. The second is that the mice are well-studied at every possible age and that a sufficient number of phenotypes were identified. Although none of the mouse models follows these rules completely, some are close. The AD50 will certainly need to be recalculated frequently in the future. Additionally, the polyQ protein aggregates phenotypes were not used for these analyses since the 600 records related to aggregates formation constitute approximately one third of the data reported in the data table. The inclusion of these data would shift the AD50 balance to a value that demonstrates the progression of aggregate formation. A very important parameter of disease progression is the lifespan of the model animals. Table 5 presents both the lifespan and the AD50 for selected models. Figure 5 additionally demonstrates that progression of the disease can be presented on the graph by plotting the increasing (cumulative) number of phenotypes against age of earliest detection. For example, Fig. 5c demonstrates progression curves and AD50 values for R6/2 and YAC128 models.

#### Neuropathology in the CNS and Non-CNS Phenotypes

A question that has frequently emerged in research on polyQ disorders is the famous “If polyQ proteins are widely expressed in virtually every cell in an organism, why are neurons in the CNS particularly vulnerable to polyQ-mediated toxicity?” To examine the issue of CNS versus non-CNS phenotypes more closely, we analyzed the localization of neuropathology in the CNS for the various polyQ mouse models. We selected the most important neuropathology phenotypes, namely, neuronal loss, neuronal morphology alteration, neuronal physiology alteration, glia abnormalities, retinopathy, neurogenesis impairment, and abnormal myelination. These neuropathology phenotypes comprise over 430 records that include data on brain location across mouse models. Additionally, we investigated separately the representative models of HD (Fig. 2), models of different polyQ disorders using native promoters (mouse or human) (Fig. 3), and the PrP promoter (Fig. 4; if more than one PrP models of the same disease were available, the neuropathology data were combined for these models). The figures depict the locations of neuropathology using diagrams of a sagittal section of the mouse brain, the spinal cord, and the eye; the brain regions that were affected by neuropathology are marked with colors. This analysis indicates that although the neuropathological phenotypes in polyQ mouse models cover many different CNS regions, they share some similarities. The profile of the neuropathology associated with any given mouse model is influenced by the promoter used for transgene expression.

The HD models are very well studied and probably also for this reason they present the broadest spectrum of brain regions and tissues that are affected by neuropathology. The main brain regions listed in our data table for HD models are obviously the striatum and the cerebral cortex. Both the R6/2 and YAC128 models also show the involvement of the hippocampus listed as neurogenesis impairment. Other brain regions and tissues involved in R6/2 are the hypothalamus, cerebellum, spinal cord, and eye retina. The Hdh(CAG)150 knock-in model does not have any of the selected neuropathologies associated with the cerebral cortex. In this model, NIIs are detected in brain regions across the brain and in the cerebral cortex. With the exception of SBMA, the consistent involvement of cerebellar neuropathology in models of other polyQ disorders is clear regardless of whether a native or non-native (such as PrP) promoter is used. The SCA3 MJD84.2 and DRPLA Q129 models with native promoters present broader spectrum of brain regions involved. The MJD84.2 reveals neuropathology in the cerebral cortex, pons, midbrain, and cerebellum that resembles the human pathology. The neuropathology map of PrP models (Fig. 4) depicts the involvement of striatum in PrP mouse models of SCA3. The neuropathology in this brain region has also been observed in patients [263–265]. The DRPLA Q129 model exhibits neuropathology in the cerebral cortex, pallidum, hippocampus, medulla oblongata, and cerebellum; this pattern of neuropathology is unique and is not present in other disorders and models (Fig. 2). The SCA1 154Q and SCA7 266Q models show the involvement of both the cerebellum and the hippocampus (Fig. 3), and this hippocampal neuropathology is also mapped in several HD models (Fig. 2).

Because HD is modeled in mice so extensively, we also asked how the HD mouse models compare to the human pathology. The use of MRI [266, 267] to image and investigate inflammatory processes in HD brains [268] demonstrates that the disease affects nearly the entire brain in patient. The specific brain regions that are affected comprise many lobes of the cerebral cortex, the caudate, putamen, globus pallidus, red nucleus, substantia nigra, thalamus, hippocampus, amygdala, brain stem, and cerebellum. Many of these brain regions are equivalent to sites of neuropathology in HD models. The models also show polyQ aggregates and brain morphology alterations that are detected in an even broader spectrum of regions and tissues. Taken together, the coverage of HD in mouse brain regions nicely matches the coverage in the human brain.

Furthermore, our data table shows that defects in the brain and the CNS do not necessarily result in the most severe phenotypes (such as a shortened lifespan). For example, mouse models with mutant polyQ expression that is selectively localized to the PCs or the striatum do not have life-threatening phenotypes. Instead, the data table shows that there are many phenotypes that are characteristic for other organs and tissues outside of the brain or the CNS. Table 6 demonstrates that NIIs are present in the vast majority of non-CNS tissues, such as the endocrine tissues (e.g., pancreas, adrenal glands, and testes), skeletal muscle, heart, lungs, liver, kidneys, skin, stomach, and duodenum. Although it is presently unclear whether these NIIs contribute to the pathogenesis, they are certainly a sign of that the disease is present in tissues other than the CNS.

**Table 6:** The non-CNS phenotypes in poly Q diseases mouse models cover a vast majority of tissues

Tissue/organ	Phenotype	Mouse models	References
Adipose tissue	Adipose tissue dysfunction	BACHD,CAG140, N171-82Q-81, R6/2	[67, 294, 302, 337, 368]
	PolyQ protein aggregates	R6/2	[34]
Adrenal gland	Hormonal dysregulation	R6/2	[67]
	Organ pathology	R6/2	[67]
	PolyQ protein aggregates	Hdh(CAG)150, R6/2	[34, 349]
Bones	Organ pathology	R6/2	[67]
Gastrointestinal tract	Hormonal dysregulation	R6/2	[258]
	Organ pathology	PrP-SCA7-c92Q, R6/2	[183, 258]
	PolyQ protein aggregates	Hdh(CAG)150, R6/2	[34, 349]
Heart	Muscle abnormalities	R6/2	[349]
	Organ pathology	BACHD, R6/2, YAC128	[68, 87, 351]
	polyQ protein aggregates	AR-97Q, R6/2	[68, 212]
Immune system	Increased level of cytokines	Hdh(CAG)150, R6/2, YAC128	[269]
Inner ear	PolyQ protein aggregates	Hdh(CAG)150, R6/2	[319]
Kidneys	Organ pathology	Hdh(CAG)150, R6/2, YAC128	[34, 87, 349]
	PolyQ protein aggregates	Hdh(CAG)150, R6/2	[34, 349]
Liver	Metabolism alteration	R6/2	[308]
	Organ pathology	R6/2, YAC128	[87, 298]
	PolyQ protein aggregates	AR100, Hdh(CAG)150, R6/2	[34, 204, 349]
Lung	Organ pathology	AR239Q	[203]
Pancreas	Hormonal dysregulation	R6/2	[67, 272]
	Organ pathology	N171-82Q-81, R6/1, R6/2	[65, 307, 325]
	PolyQ protein aggregates	AR-97Q, AR239Q, Hdh(CAG)150,N171-82Q-81, R6/1, R6/2	[34, 203, 212, 307, 325, 349]
	Diabetes mellitus	BACHD, Hdh(CAG)150, N171-82Q-81, R6/1, R6/2	[77, 272, 302, 307, 357]
Peripheral nervous system	Neuronal morphology alterations	PrP-SCA7-c92Q, R6/2	[183, 364]
	Neuronal loss	PrP-SCA7-c92Q	[183]
	PolyQ protein aggregates	PrP-SCA7-c92Q	[183]
	Abnormal myelination	MJD84.2	[164]
Skeletal muscle	Muscle abnormalities	AR-97Q, AR100, AR113Q, AR120, Hdh(CAG)150, HSA-AR-141, NLS-N171-82Q, R6/1, R6/2	[34, 204, 209, 211, 212, 217, 281, 298, 345, 346, 349]
	PolyQ protein aggregates	AR-97Q, AR100, AR113Q, Hdh(CAG)150, HSA-AR-141, R6/2	[34, 204, 208, 212, 217, 349]
Skin	PolyQ protein aggregates	AR239Q	[203]
Spleen	Organ pathology	YAC128	[87]
Testes	Fertility	AR120, Q71-B, R6/2	[157, 211, 335]

We also retrieved data regarding non-CNS phenotypes other than NIIs that are present in the polyQ mouse models (Table 6). A detailed list of non-CNS phenotypes is provided in the data table and includes changes in organs such as the heart, kidneys, liver, pancreas, spleen, stomach, testes, bones, pituitary gland, skeletal muscle, epididymis, brown fat, adrenal glands, and peripheral nervous system. This list clearly indicates that many organs and systems are affected in polyQ diseases. The majority of non-CNS organs and tissues that exhibit pathology show, e.g., organ enlargement, altered morphology, muscle abnormalities, and many other features. This finding may indicate that the most severe phenotypes that lead to the deaths of these transgenic model animals are located outside of the brain and CNS. One exciting finding is the demonstration of immune system involvement in mouse models of HD [269]. The R6/2, Hdh(CAG)150, and YAC128 model animals show increased levels of serum IL-6 and other cytokines, along with excessive activation of stimulated monocytes, macrophages, and microglia [270]. Similar findings on the contribution of immune system were confirmed in HD patients [270]. Another issue is that the dysfunction of neurons that are located outside of the brain or the CNS may lead to extremely severe phenotypes. Gastrointestinal abnormalities in the PrP-SCA7-c92Q mouse are related to the degeneration of enteric neurons and may contribute to decreased lifespan in this model [183]. Another example is the SCA1 154Q knock-in mouse, which has breath-control abnormalities that probably lead to death [149], and this phenotype may well be related to defects in neurons that are not directly located in the brain but in the nuclei of the medulla.

Therefore, we should not ask “Why the brain?” or “Why the CNS?” Rather, we should analyze the broader context of polyQ disorders, wherein pathogenesis may be located throughout the organs and tissues of the entire body. Therefore, we should ask, “Which tissues and why these tissues?,” “Why neurons and why these particular neurons?,” and “Why neurons outside the CNS?”

## Electronic supplementary materials

Below is the link to the electronic supplementary material.

ESM 1 (DOCX 29 kb)

ESM 2 (XLSX 431 kb)

ESM 3 (JPEG 28 kb)

High resolution image (TIFF 2198 kb)

ESM 4 (JPEG 46 kb)

High resolution image (TIFF 2326 kb)

ESM 5 (JPEG 19 kb)

High resolution image (TIFF 1301 kb)
