# Supplementary material for: Mouse Models of Polyglutamine Diseases: Review and Data Table. Part I
Source: Mol Neurobiol. 2012 Sep 7;46(2):393–429. doi: 10.1007/s12035-012-8315-4 (PMC3461215; doi:10.1007/s12035-012-8315-4)
Supplement: Supplementary file 1 — (DOCX 29 kb) [file 12035_2012_8315_MOESM1_ESM.docx]

# Supplementary information

## Table design

The data table is created in Excel and we have selected this application since it is intuitive and well accessible to broad scientific audience that mostly need no introduction to immediately start work with such data table. We concluded that advantage of accessibility, ease of use and the possibility of self-modification prevail over the obvious drawbacks of excel based lists. For this data table we have carefully analyzed research reports describing polyQ mouse models and selected ~240 articles presenting the most current and reliable information. The reason for selecting only a pool of research work for the data table was that some reports presented contradictory data and also redundant data. Therefore where possible we were searching for second or third report confirming the given phenotype but our selection was still arbitrary. Additionally we did not include all works that report a certain phenotype to preserve transparency which makes the straight forward comparison of models possible. Our data table is not the database but when necessary it can be converted to SQL-type databases files by the end user. Moreover our data table possesses many features of a database and the sets of defined information can be easily retrieved using either filter functions or pivot tables and pivot charts.

The phenotypes in polyQ mouse models are very different and hard to compare. Therefore to categorize the phenotypes in polyQ diseases we first established the system that aided the systematic construction of our tables and later helped to retrieve the data to compare mouse models. Constructing the system for the table we have also used some phenotype names from the Mammalian Phenotype Browser available at <http://www.informatics.jax.org>. Our system is composed of three elements namely (I) the columns describing the phenotype, (II) when applicable the columns describing the location where the phenotypes were detected in CNS or other tissues, and (III) the numeric part that consists of two phenotype time points called “age of earliest detection (AGE I, weeks)” and “age of latest detection (AGE II, weeks)” expressed in weeks and column named % change that contains data on decrease or increase of the phenotype versus WT animal.

(I) The columns should describe the phenotype in most defined way and simultaneously allow for easy sorting and data analysis. To accomplish those conditions we have concluded that phenotypes must be described by 3 columns with increasing detail content. First column “general phenotype” divides the phenotypes to only 4 categories: motor (M), neuropathological (N), cognitive (C) and other (O). Second column “phenotype” gives another level of complexity such as for instance “balance and coordination alterations” or “neuronal morphology alterations”. The cells in third column called “detailed phenotype” contain the most detailed signs such as “rotarod impairment”, “brain volume decrease”, “heterotopic purkinje cells”, “dendritic degeneration”. Such phenotype description facilitates the sorting of phenotypes that can be accomplished by all three columns.

(II) Another two columns describe the location where the phenotypes were detected in CNS or other tissues. The column called “CNS region” describes the region – location such as “striatum” or “cerebellum” and the column “tissue, cells, organ” adds more details such as “Medium spiny neurons” or “Purkinje cells”. The column “tissue, cells, organ” also holds information if the phenotype was located outside of the CNS such as “lung” or “muscles”.

(III) The numeric columns contain “age of earliest detection” that is reflecting the earliest age of mice in weeks when the phenotype was first noticed. The “age of latest detection” contains the latest age of mice in weeks when the phenotype was last examined by the researchers. Detailed description of how the time points were listed is provided in Supplementary Table 1. Additionally, in the time point of phenotype “polyQ aggregates” sometimes the arbitrary threshold has been applied. This threshold was set to either “moderate” number of inclusions or ++ as reported by various research works.

Recording the time points of phenotypes in this dual system is giving the right balance between the information transparency and recorded details. The very special case is the “decreased lifespan” phenotype that records the death of 10% of mice in the cohort as “age of earliest detection” and the death of 90% of mice in the cohort as “age of latest detection” however it was not always possible to apply such threshold. In addition the surrogate measures of lifespan that are in accordance with local animal welfare regulations may lead to differences in estimation. The mean values for “decreased lifespan” are noted in the commentaries column. The column % change contain quantification data from the original publication. Moreover some quantification data from research reports were processed not only by reading and collecting data but also literally “manually” by reading the published graphs with scales where possible.

## The methods of retrieving the information from the data table

The data table is constructed in Excel and therefore uses its common built-in filter functions for the most basic data retrieval and comparison. The filters are accessible from the first row of the Excel sheet allowing the selection of the criteria from drop-down lists or applying a sorting. The first “number” column holds the record numbers that allow for restoring the initial order of records. The data table can be filtered and sorted to retrieve the data of interest and this can be done in many different scenarios. First and the simplest scenario involve the retrieving of the information for known single mouse model or many mouse models from single disease. Since some of the filter dropdown lists are long we recommend starting the information retrieval using the columns with fewer criteria. These are: “disease” (9 diseases), “model type” (8 types) and 4 “general phenotype” (cognitive, motor, neurological, other). Therefore for instance finding the SCA3 YAC from Cemal et al. 2002 model involves making selection in “disease” and “model type” columns. The example of slightly more complicated question would be to find mouse models that exhibit NIIs in deep cerebellar nuclei and find out when these NIIs are formed. Although more direct way is also possible, it is again more comfortable to start from the columns with less criteria and select “neuropathological” from “general phenotype” column, than “cerebellum” from “CNS region” column followed by selecting “polyQ protein aggregates” from “phenotype” column, than selecting the “nuclear inclusions” from “detailed phenotype”, and values that include “dentate nucleus” or “deep cerebellar nuclei” phrases from “detailed localization” column. In result we will get three DRPLA mouse models (Sato et al., 2009, Ying et al., 2006, Schilling et al., 1999), seven SCA3 mouse models (Cemal et al. 2002, Goti et al., 2004, Bichelmeier et al., 2007, Chou et al., 2008 ) and N171-82Q model having the inclusions in deep cerebellar nuclei. The two strategies described above can be combined and modified to retrieve more complicated results or compare multiple mouse models.

To perform more complicated data search and comparison we recommend using pivot tables and pivot charts that are one of the most powerful tools in Excel. The pivot tables were made to use with the numeric data but it can also be used for grouping and integrating text data from multiple columns of spreadsheet into one column in pivot table that creates tree-like structure allowing for uncovering the relation between the data. The easiest example is integrating two spreadsheet columns such as “disease” and “practical model name”. After using the function “insert pivot table” and selecting columns to be integrated, the pivot table will display list of mouse models for every disease. Additionally it is possible to apply filter in pivot table and for example display only HD and SCA3 models. Above example integrates only 2 columns but it is possible to construct research strategies integrating 4 or more columns into one pivot table column. Such strategy combined with filtering can uncover quite complicated relation of data and is similar to querying the database. The relations and datasets can subsequently be retrieved to separate table or spreadsheet for further processing. The pivot table can also be used for all operations on the data table where counting of records is important and to do this it is sufficient to drag the required column into the numeric field of pivot table. This strategy combined with the tree-like structure of text data and filters allows for finding numeric relation in text data. To illustrate the data retrieval from pivot table we have created a pivot table located in a separate sheet in our data table file.

## Supplementary tables and figure captions

Supp. Table 1. The names of columns in the data table I and the short description of data content in each column.

| **Columns** | **Description** |
| --- | --- |
| ***number*** | The record number |
| ***disease*** | The disease that is modeled by the mouse model |
| ***ref:first author*** | The first author of the referenced work |
| ***ref:PMID*** | The PMID number of the referenced work (contains appropriate PUBMED link) |
| ***ref:year*** | The year of the referenced work |
| ***practical name/strains*** | The name of the mouse that was used in the original publication |
| ***model type*** | The model type comprises random integration models that can be Transgenic, YAC, BAC or cosmid transgenic as well as Targeted (knock-in) and Transgenic (conditional) |
| ***TC:promoter*** | The promoter used in transgenic construct (TC) that also drives the expression in mice |
| ***TC:coding seq***  ***/inserted seq*** | Short information about the coding sequence used for transgenesis or the information about inserted sequence in knock-in models |
| ***TC:Q number*** | The number of CAG repeats or number of glutamines (Q) present in transgenic mouse |
| ***genetic background*** | The genetic background of mice, listed using the nomenclature from  “Guidelines for Nomenclature of Mouse and Rat Strains” (<http://www.informatics.jax.org/nomen/strains.shtml>) with minor changes:  mixed complex mixed background  strain1;strain2 mixed background  strain1(Nx);strain2 mixed background, backcrossed less than 5 times (x) onto strain1  strain1(Nx).strain2 incipient congenic strain, backcrossed 5-9 times (x) onto strain1  strain1 congenic strain (at least 10 backcrosses onto strain1)  (strain1 x strain2)F1 F1 hybrids created by intercrossing congenic strain1 and strain2  not specified not mentioned in the publication or unclear |
| ***general phenotype*** | The phenotypes in this column were divided into four categories: motor (M), neuropathological (N), cognitive (C) and other (O). The general phenotypes are color-coded |
| ***phenotype*** | Contains the 43 groups of phenotypes |
| ***detailed phenotype*** | This column lists 195 detailed phenotypes that fall into 4 categories of general phenotypes and 43 phenotypes |
| ***evaluation method*** | General description of method used for detection of detailed phenotypes |
| ***CNS region*** | This column lists central nervous system regions where the “detailed phenotype” is detected |
| ***non-CNS region*** | This column lists other tissues in mouse body where the “detailed phenotype” is detected |
| ***detailed localization*** | This column refines and adds more details (additional tissue type or cell type) to both “CNS region” and “non-CNS region” – locations of “detailed phenotype” |
| ***age of earliest detection (AGE I, weeks)*** | This column lists the time points of “detailed phenotype”. If the phenotypes were subjected to statistical analyses (behavioral tests: rotarod, open field, cognitive tests) only the earliest significant time point was listed. If the phenotype has a simple non-statistical nature (present/not present; such as “dark cell degeneration”) earliest time point of observation is listed. For the the phenotype “nuclear inclusions” the arbitrary threshold has been applied. |
| ***age of latest detection (AGE II, weeks)*** | This column lists the time points of phenotypes that were reported as the last examination of phenotype that was significant or as above (present/not present) |
| ***% change*** | If the phenotypes were quantified and if the data is available this column lists the percent of phenotype change (decrease or increase) in transgenic versus non transgenic animals. If the parameter is decreased the value contains the minus sign. Additionally the column is color coded (red table cells for increase, blue table cells for decrease);. |
| ***comments*** | various comments |

The supplementary figures show the basic statistics and basic numbers related to the data table.

Supp Fig 1. The summary of data in the data table I showing the number of models, “phenotypes” and “detailed phenotypes” in relation to the particular polyQ diseases. The majority of mouse models and phenotypes were generated and detected in HD which is most extensively studied by researchers.

Supp Fig 2. The diagram shows the summary of the number of “detailed phenotypes” identified in the mouse models of polyQ disorders. The best characterized animals have an arbitrary range of 170 (R6/2 without variants) to 20 phenotypes. The “gold standard” in characterization of pQ disease models is the R6/2 mouse.

Supp Fig 3. The summary of the data table showing promoters used in constructing the disease mouse models. The PrP, PcP2 and different types of HD promoters are most intensively used for modeling polyQ disorders.
