# Supplementary figures and images for: Mouse Models of Polyglutamine Diseases: Review and Data Table. Part I
Source: Mol Neurobiol. 2012 Sep 7;46(2):393–429. doi: 10.1007/s12035-012-8315-4 (PMC3461215; doi:10.1007/s12035-012-8315-4)

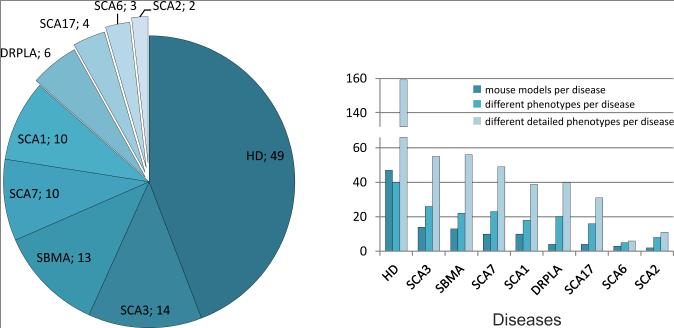

Supplement: Supplementary file 3 — (JPEG 28 kb) [file 12035_2012_8315_Fig6_ESM.jpg]

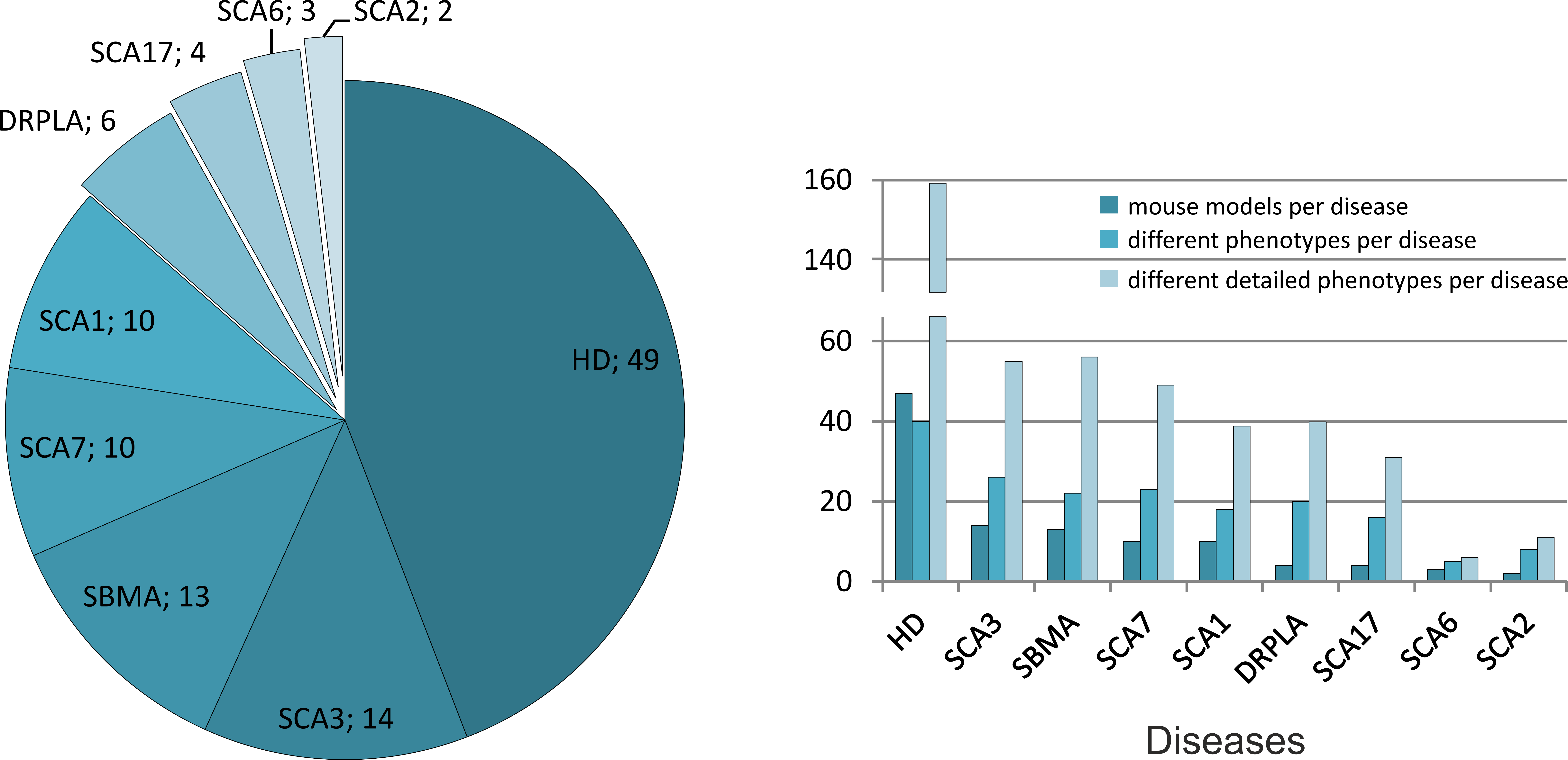

Supplement: Supplementary file 4 — High resolution image (TIFF 2198 kb) [file 12035_2012_8315_MOESM3_ESM.tif]

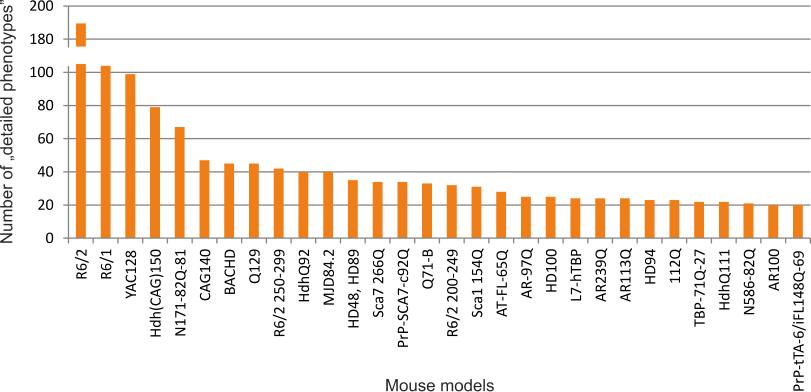

Supplement: Supplementary file 5 — (JPEG 46 kb) [file 12035_2012_8315_Fig7_ESM.jpg]

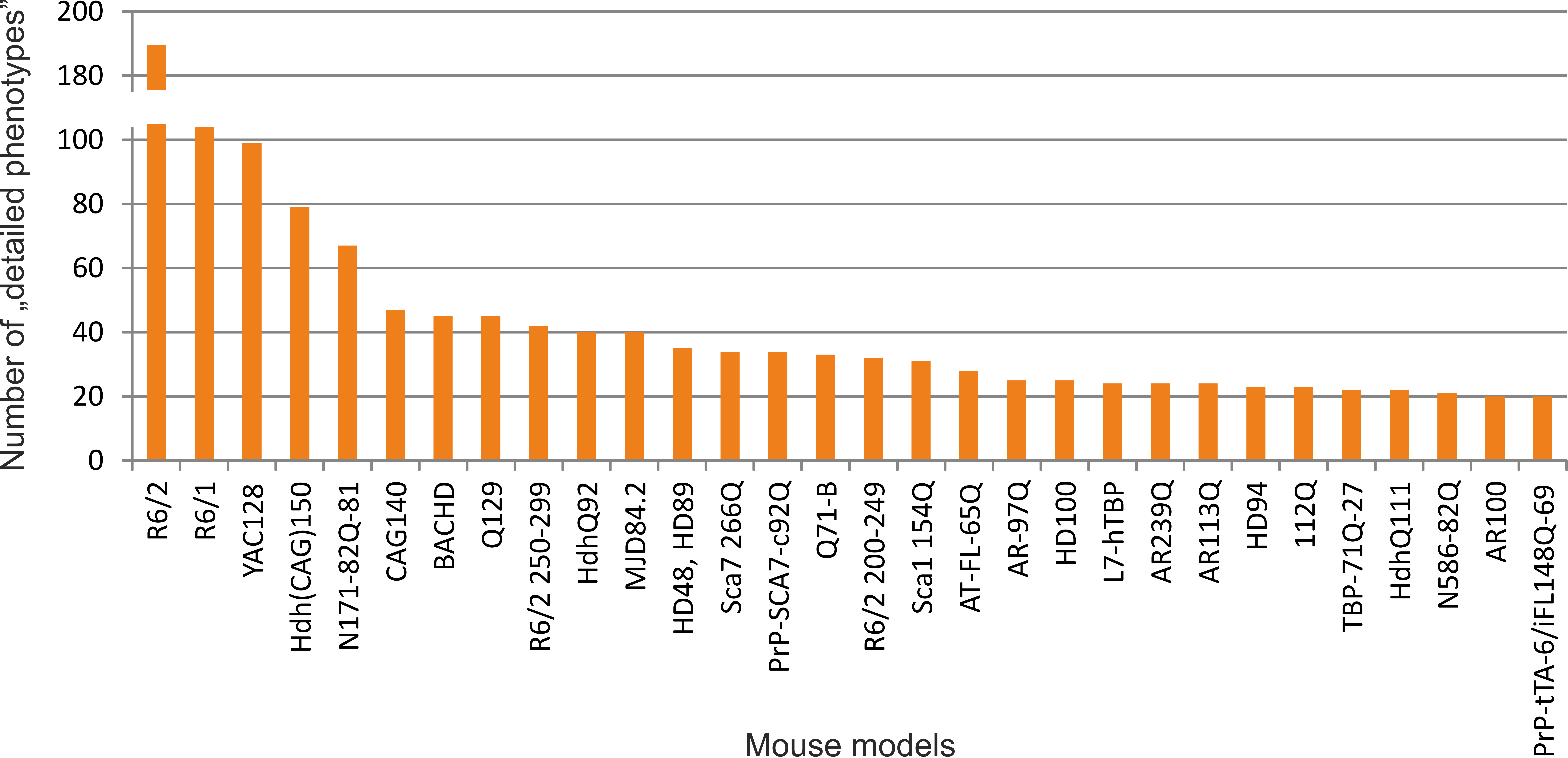

Supplement: Supplementary file 6 — High resolution image (TIFF 2326 kb) [file 12035_2012_8315_MOESM4_ESM.tif]

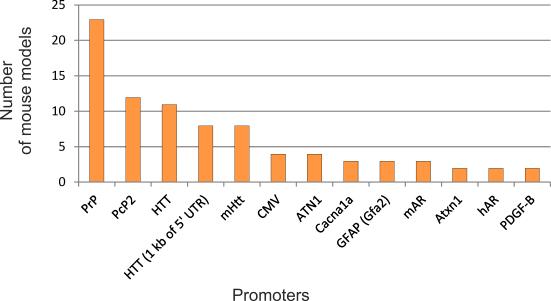

Supplement: Supplementary file 7 — (JPEG 19 kb) [file 12035_2012_8315_Fig8_ESM.jpg]

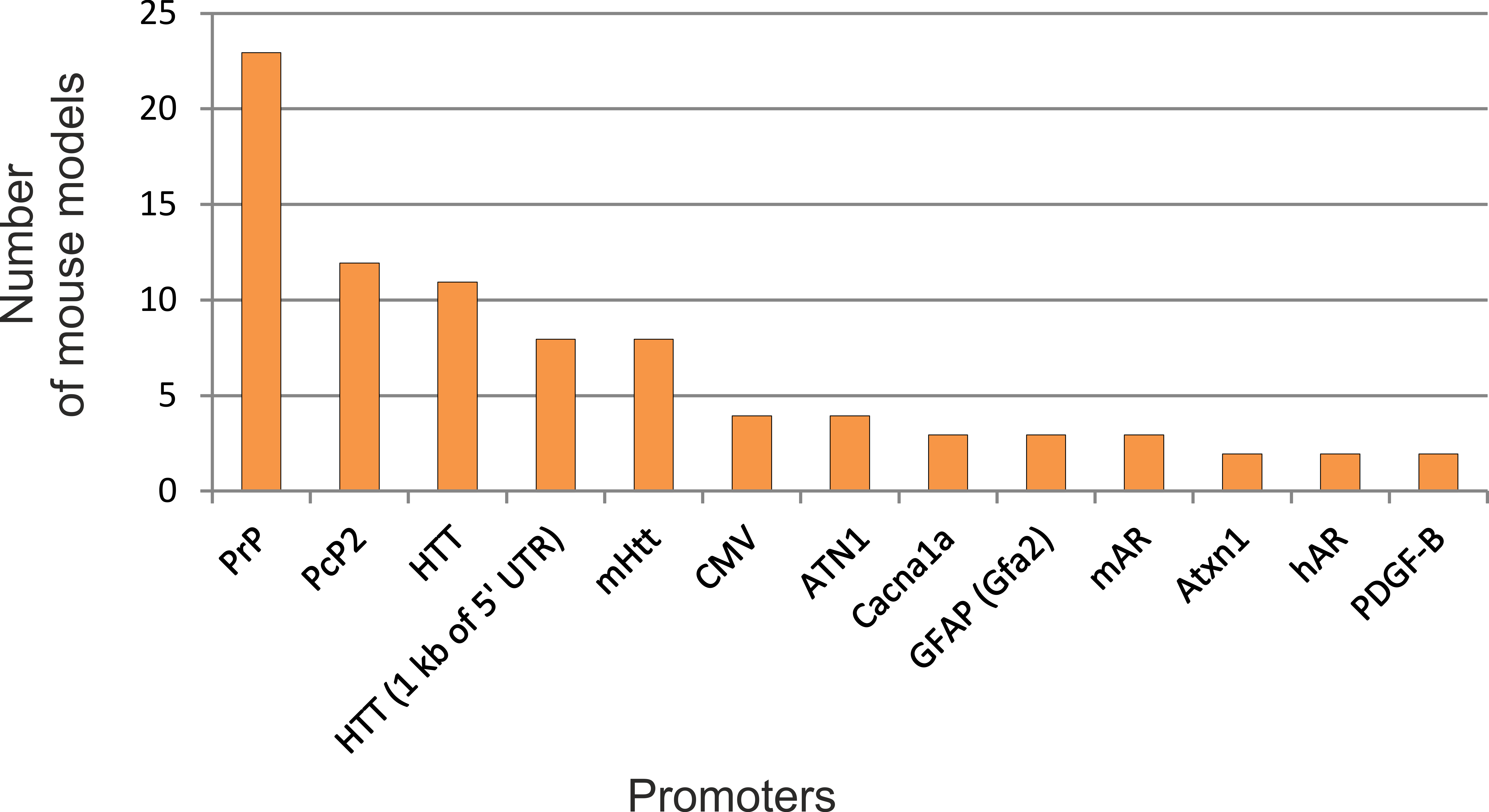

Supplement: Supplementary file 8 — High resolution image (TIFF 1301 kb) [file 12035_2012_8315_MOESM5_ESM.tif]
